# Identifying C1QB, ITGAM, and ITGB2 as potential diagnostic candidate genes for diabetic nephropathy using bioinformatics analysis

**DOI:** 10.7717/peerj.15437

**Published:** 2023-05-25

**Authors:** Yongzheng Hu, Yani Yu, Hui Dong, Wei Jiang

**Affiliations:** 1 Department of Nephrology, The Affiliated Hospital of Qingdao University, Qingdao, Shandong, China; 2 Health Management Center, The Affiliated Hospital of Qingdao University, Qingdao, Shandong, China

**Keywords:** Diabetic nephropathy, Bioinformatics analysis, Microarray, RNA regulatory pathways, qPCR, Key gene

## Abstract

**Background:**

Diabetic nephropathy (DN), the most intractable complication in diabetes patients, can lead to proteinuria and progressive reduction of glomerular filtration rate (GFR), which seriously affects the quality of life of patients and is associated with high mortality. However, the lack of accurate key candidate genes makes diagnosis of DN very difficult. This study aimed to identify new potential candidate genes for DN using bioinformatics, and elucidated the mechanism of DN at the cellular transcriptional level.

**Methods:**

The microarray dataset GSE30529 was downloaded from the Gene Expression Omnibus Database (GEO), and the differentially expressed genes (DEGs) were screened by R software. We used Gene Ontology (GO), gene set enrichment analysis (GSEA), and Kyoto Encyclopedia of Genes and Genomes (KEGG) pathway enrichment analysis to identify the signal pathways and genes. Protein-protein interaction (PPI) networks were constructed using the STRING database. The GSE30122 dataset was selected as the validation set. Receiver operating characteristic (ROC) curves were applied to evaluate the predictive value of genes. An area under curve (AUC) greater than 0.85 was considered to be of high diagnostic value. Several online databases were used to predict miRNAs and transcription factors (TFs) capable of binding hub genes. Cytoscape was used for constructing a miRNA-mRNA-TF network. The online database ‘nephroseq’ predicted the correlation between genes and kidney function. The serum level of creatinine, BUN, and albumin, and the urinary protein/creatinine ratio of the DN rat model were detected. The expression of hub genes was further verified through qPCR. Data were analyzed statistically using Student’s t-test by the ‘ggpubr’ package.

**Results:**

A total of 463 DEGs were identified from GSE30529. According to enrichment analysis, DEGs were mainly enriched in the immune response, coagulation cascades, and cytokine signaling pathways. Twenty hub genes with the highest connectivity and several gene cluster modules were ensured using Cytoscape. Five high diagnostic hub genes were selected and verified by GSE30122. The MiRNA-mRNA-TF network suggested a potential RNA regulatory relationship. Hub gene expression was positively correlated with kidney injury. The level of serum creatinine and BUN in the DN group was higher than in the control group (unpaired t test, *t* = 3.391, *df* = 4, *p* = 0.0275, *r* = 0.861). Meanwhile, the DN group had a higher urinary protein/creatinine ratio (unpaired t test, *t* = 17.23, *df* = 16, *p* < 0.001, *r* = 0.974). QPCR results showed that the potential candidate genes for DN diagnosis included C1QB, ITGAM, and ITGB2.

**Conclusions:**

We identified C1QB, ITGAM and ITGB2 as potential candidate genes for DN diagnosis and therapy and provided insight into the mechanisms of DN development at transcriptome level. We further completed the construction of miRNA-mRNA-TF network to propose potential RNA regulatory pathways adjusting disease progression in DN.

## Introduction

Diabetic nephropathy (DN) is a serious complication caused by diabetic microangiopathy, which often progresses to end-stage renal disease (Singh, Winocour & Farrington, 2011). The pathological changes in DN included glomerular vascular injury, glomerulosclerosis, nodular lesion formation, renal function deterioration, and, ultimately, end-stage renal disease (Qi et al., 2017). Globally, 30 to 40% of diabetes cases have been reported to progress to DN. In spite of substantial advances in DN research in recent years, a large proportion of patients still irreversibly suffer from end-stage renal disease. Compared to other complications, metabolic disorders caused by DN make it more difficult to treat end-stage renal disease once they develop. Therefore, early diagnosis and timely intervention in DN are becoming more and more important (Nakagawa et al., 2011; Ruiz-Ortega et al., 2020).

The vast amounts of data generated by new technologies such as genome sequencing and microarray chips have expanded the ways in which data can be analyzed and interpreted for biological understanding and therapeutic advances (Bednar, 2000). However, most resent studies on DN have focused on exploring the expression of differential genes that affect glomerular pathological changes. These cannot fully reflect the pathogenesis of DN, because in the context of diabetes, renal tubular function plays an important role in regulating glomerular filtration (Vallon & Thomson, 2012). However, it is not appropriate to focus on the single transcription level of mRNA (Gao et al., 2021; Chen et al., 2022), and attention should be paid to other components in the transcriptional regulatory network.

MicroRNA (miRNA) are a class of endogenous small RNA with a length of about 20–24 nucleotides that play a variety of important regulatory roles in cells (Wang, Chen & Sen, 2016). MiRNA regulate gene expression mostly by degrading mRNA (Etheridge et al., 2011). In recent years, new progress has been made in the study of miRNA and human diabetes (Vasu et al., 2019).

Transcription factors (TFs) can activate or suppress gene transcription by binding to specific parts of chromatin. Previous studies mostly focused on the relationship between lncRNA-miRNA-mRNA in ceRNA networks (Guo et al., 2022), but TFs are also involved in complex transcriptional regulation. TF expression is closely related to the physiological and pathological state of cells, and their expression levels in tissues are specific in time and space. Therefore, changes in TF expression reflect changes in cell state and may lead to pathological processes (Lambert et al., 2018) such as diabetes (Polvani et al., 2016). However, in-depth research has revealed that TFs function through complex regulatory patterns, even though TFs in the same family may have a bidirectional effect on the progression of the same disease (Song et al., 2016; Rad et al., 2016). Therefore, it is crucial to accurately explore the role of both miRNA, and TFs in DN. Further understanding of the direct regulatory relationship between mRNA, miRNA and TF can provide new insights for personalized disease management, diagnosis and prognosis.

In this study, we selected the GSE30529 dataset containing DN renal tubule samples from the GEO database, used the robust multiple array averaging (RMA) method in R language to preprocess and normalize the data, and identified differentially expressed genes (DEGs) according to screening criteria. After that, gene set enrichment analysis (GSEA), Gene Ontology (GO), and Kyoto Encyclopedia of Genes and Genomes (KEGG) analysis were applied to identify the biological processes, cellular components and molecular functions in which the DEGs were involved. Subsequently, a relevant protein-protein interaction (PPI) network was constructed, and the MCC algorithm of Cytohubba plug-in in Cytoscape software was used to identify the top 20 genes with the highest interaction scores as hub genes. Next, based on the GSE30122 dataset containing 69 samples, the ROC method was used to verify those genes. Subsequently, five Hub-genes with the most potential diagnostic values were selected. MiRNA and TF targets were predicted through four different online prediction websites, and the ‘nephroseq’ database was used to predict the correlation between genes and kidney function. The miRNA-mRNA-TF interaction was completed to elucidate the mechanism by which these genes and expression elements interact and cooperate to drive the occurrence and development of DN. Finally, the expression of hub genes in DN rats was further verified using qPCR. Altogether, these results have the potential to elucidate novel candidate genes associated with DN and provide a new insight into its molecular basis.

## Materials & Methods

### Microarray data acquisition

**Figure 1:**
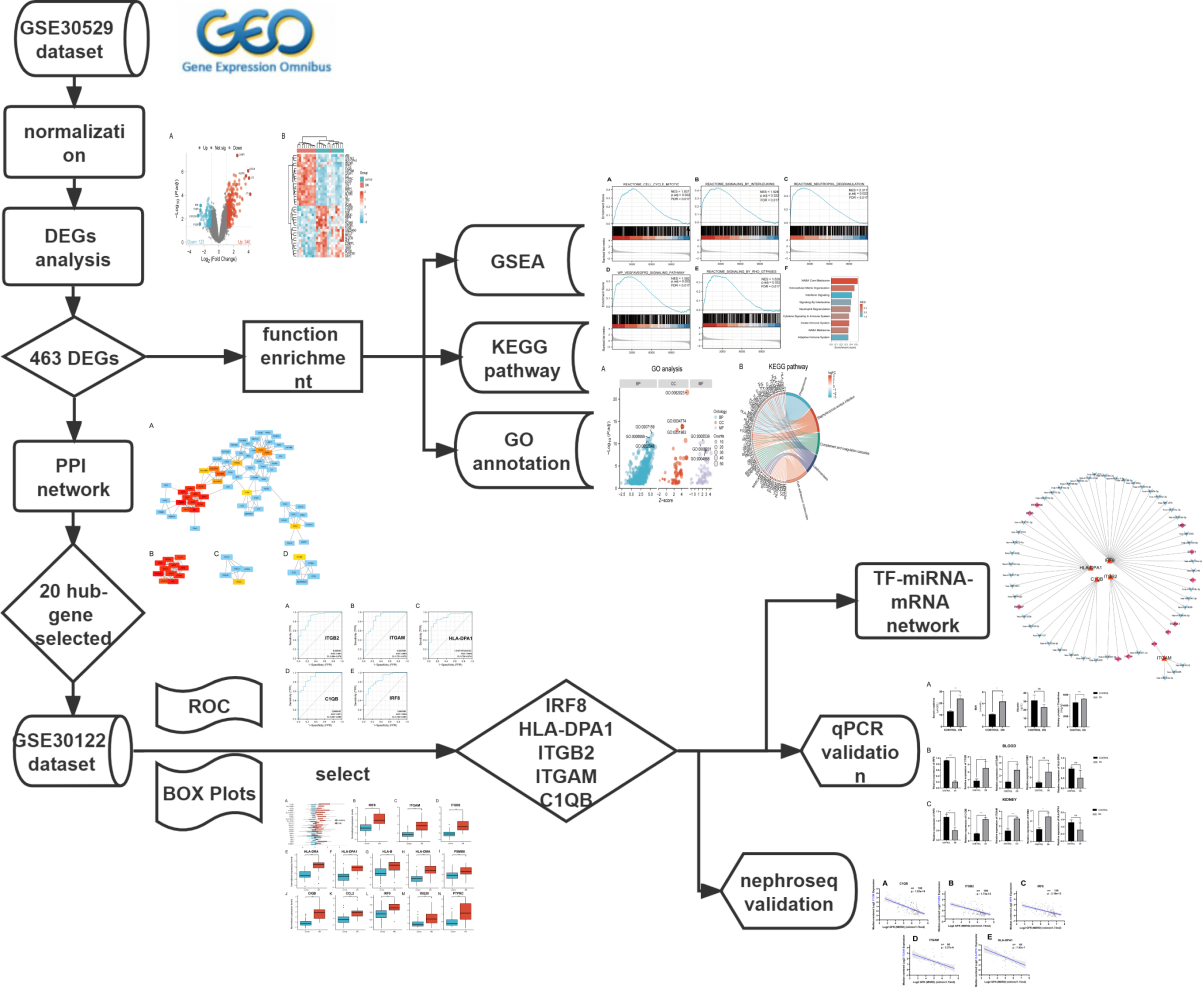
The workflow of the study. DEGs, differentially expressed genes; DN, diabetic nephropathy; GSEA, gene set enrichment analysis; GO, gene ontology; KEGG, Kyoto Encyclopedia of Genes and Genomes. PPI, protein-protein interaction Networks; ROC, receiver operating characteristic curve; AUC, area under curve; TF, transcription factors.

The mRNA expression profile datasets were retrieved from the GEO database (https://www.ncbi.nlm.nih.gov/geo/). We uesd the keywords “Diabetic Nephropathy,” “Homo sapiens,” and “High throughput gene expression profile” as search criteria, and the datasets GSE30529 and GSE30122 fit the above screening conditions. Both GSE30529 and GSE30122 were located on the GPL571 platform ((HG-U133A_2) Affymetrix Human Genome U133A 2.0 Array). GSE30529 was used to screen for differential genes associated with DN, while GSE30122 was used to verify and explore the predictive value of genes. GSE30529 included 10 diabetic tubule samples and 12 control samples to investigate the influence of diabetes mellitus on DEGs. GSE30122 contained nine glomeruli and 10 tubule samples from kidneys with DN and 50 control glomeruli samples from the unaffected portion of tumor nephrectomies. The Series Matrix files for two datasets including gene expression counts were downloaded for downstream analysis. Gene ID conversion based on the genome “hg38” was performed on the count matrix, the differential expression values of the same gene were averaged, and the original data were log2 transformed and normalized. A workflow of our study is displayed in Fig. 1.

### Analysis of differential gene expression

The ‘boxplot’ software package was used to draw the box diagram to evaluate the distribution of the sample expression matrix, and the annotation file of the dataset was used to complete the annotation of the probe. Principal component analysis (PCA) was used to verify the reproducibility of the data. The ‘Limma’ package (version 3.42.2) in R software was used to screen the differential genes. The criteria for DEGs were an adjusted *P* value less than 0.05 and absolute fold change (FC) greater than 1.0. Heatmaps and volcano plots were used to visualize these DEGs. Box line plots, PCA, heat map and volcano maps were created using the ‘ComplexHeatmap’ (version 2.2.0) and ‘ggplot2’ packages (version 3.3.3) in R (version 3.6.3) (Gu, Eils & Schlesner, 2016).

### GSEA

GSEA was used to assess gene distribution trends and explore DEGs related phenotypes in DN (Subramanian et al., 2005). The ‘clusterProfile’ package (Yu et al., 2012) in R and C2: curated gene set (c2.cp.v7.2.symbols.gmt) were used for functional enrichment analyses. An absolute net enrichment score (NES) > 1, false discovery rate (FDR) *q* < 0.25, and *P* value < 0.05 were considered to represent significant enrichment.

### GO and KEGG pathway enrichment analyzes of genes

The R software clusterProfler package was also used to analyze the GO enrichment and for the KEGG pathway and Reactome enrichment analyses. The species was limited to Homo sapiens. The GO terms covered three aspects: molecular function (MF), cellular component (CC), and biological process (BP). Enrichment results were visualized using a circle plot and a chord plot. The significant enriched functions and pathways were selected with an adjusted *p* value < 0.05.

### PPI analysis and correlation analysis of DEGs

The PPI network was completed using the online tool STRING (https://string-db.org/) constructed on all DEGs, with filtering conditions (combined score > 0.4). Next, we downloaded the interaction information and optimized PPI networks using Cytoscape software (v3.9.1) to improve visualization. We used the Minimal Common Oncology Data Elements (MCODE) to select crucial gene clusters and calculate cluster scores (filter criteria: degree truncation = 2; node score cut-off = 0.2; k-core = 2; max depth = 100). With the MCC algorithm of the Cytohubba plug-in used in Cytoscope, the top 20 genes with the highest scores were labeled as hub genes (Chin et al., 2014).

### Construction of the miRNA–mRNA–TF interaction network

Both miRNAs and TFs play independent or integrated roles in transcriptional regulation (Zhao et al., 2016). We predicted target miRNAs of selected genes using four systematic online miRNA databases: TargetScan, starBase, miRWalk, and miRDB. MiRNAs predicted by at least three of the databases were considered reliable. Similarly, transcriptional regulatory relationships unraveled by sentence-based text mining (TRRUST) (https://www.grnpedia.org/trrust/) was used to predict TF-mRNA interactions. Subsequently, the miRNA–mRNA–TF network was constructed by Cytoscape software.

### Clinical correlation with selected hub genes

Nephroseq (https://nephroseq.org/) is a free platform used by the academic and non-profit community for integrative data mining of genotype/phenotype data. We used the tool ‘Nephroseq V5’ to determine the correlation between selected hub genes and renal function. The expression files of two datasets containing chronic kidney disease (CKD) patients were downloaded. GraphPad Prism 9 (version 9.0.0) was used to replot the scatter plots.

### Animal models and sampling

Six male healthy Wistar rats (weighing 250–300 g) were obtained from Shandong Helix Biotechnology Co., Ltd. The rats were housed in animal laboratory of affiliated hospital of Qingdao university at 24 °C on a 12-hour light/dark cycle and were given ad libitum access to food for one week prior to the commencement of the experimental procedures. The medical ethics committee of Affiliated Hospital of Qingdao University approved all animal experiments *(QYFY-WZLL-27726)*. A simple random sampling technique was used to divide the rats into two groups. Three rats served as normal controls, while the remaining rats (DN group, *n* = 3) were administered a single intraperitoneal injection of streptozotocin (STZ) (55 mg/kg body weight, Sigma-Aldrich, St. Louis, MO, USA) in sodium citrate buffer (pH 4.5) following a 12-hour fast. Administration of an equal volume of sodium citrate buffer served as a vehicle control (control group, *n* = 3). Plasma glucose concentrations were measured from blood collected from the tail vein on three consecutive days using an AccuChek glucometer (Roche Diagnostics, Indianapolis, IN, USA) one week post-injection. The diabetic rat model was established when plasma glucose levels were ≥16.67 mmol/L. The early DN rat model was defined based on the urine protein to creatinine ratio eight weeks after the injection.The urine was collected after 24 h with a rat metabolic cage. After anesthesia with isoflurane, rats in each group were used for blood collection *via* the orbital venous plexus. At the end of the experimental protocol, all rats were sacrificed following anesthesia with isoflurane and samples (including blood and kidney) were immediately collected for further analysis. In order to reduce individual differences caused by the environment and other factors, each sample was sampled three times.

### Urine biochemical parameter analysis

The levels of urine microalbumin in each group were measured according to the manufacturer’s protocol (E-EL-R0025c; Wuhan Elabscience, Wuhan, China). The absorbance was measured at 450 nm using a Varioskan Flash™ multimode microplate reader (Thermo Fisher Scientific, Waltham, MA, USA). The relevant assay kitswere obtained to determine the levels of creatinine *via* the sarcosine oxidase method (E-BC-K188-M; Wuhan Elabscience, Wuhan, China), serum levels of BUN *via* the urease-glutamate dehydrogenase method (S03036; Rayto, Shenzhen, China), and serum levels of albumin *via* the Bromocresol green method (S03043; Rayto, Shenzhen, China).

### RT-PCR validation of hub genes

Based on the results of the above analysis, the expression levels of the five key genes were verified in blood samples and kidney samples. Each type of sample was composed of three DN rats and three control rats. Kidney tissue samples were collected and immediately snap-frozen in liquid nitrogen to preserve RNA integrity. The samples were then stored at −80 °C until further processing. Total RNA was isolated using TRIzol reagent (CW0580S; JiangSu CoWin Biotec, Beijing, China) in accordance with the manufacturer’s instructions. The isolated RNA was reverse transcribed into cDNA by Evo M-MLV RT Premix (AG11706; Accurate Biology, China). The SYBR^®^ Green Premix Pro Taq HS qPCR Kit (AG11701; Accurate Biology, China) and an ABI 7500 PCR system (ABI, Waltham, MA, USA) were used for quantitative real-time PCR analysis. The 2 −ΔΔCt method was employed to determine the relative expression of the hub gene, with normalization to the expression level of glyceraldehyde 3-phosphate dehydrogenase (GAPDH). The primer sequences are provided below: C1QB (F: 5′-TCAACAGCGCCCTGCGACCAAACCA-3′, R: 5′-TGAACTTGCCACTGCGCGGCTCGTA-3′), ITGAM (F: 5′-AAACCCGAGTGGTTGTTGCAGCCCC-3′, R: 5′- ATGGGGTCGCACCGGTTTGTGCTGT-3′), ITGB2 (F: 5′-AGCCTGCCAGCCTCCGTTTGCCTTT-3′, R: 5′- TGCATTATGGCATCCAGCCCGCCCT-3′), HLA-DPA1 (F: 5′-TTTGTGCAGACGCAGCACCCGT-3′, R: 5′- AAGAGCCTCCTGGG CGTCAAACGCA-3′), and IRF8 (F: 5′- ACCTGCAGCAGTTCTACGCCACCCA-3′, R: 5′- AGTTTGGAGCGCAAGGGCGCTGTGT-3′). All experiments were repeated three times.

### Statistics analysis

The R software ‘ggpubr’ package was used to perform statistical analyses. Unpaired Student’s *t*-test was used to compare the mean differences in the PCR results and biochemical indexes, while the Mann–Whitney U test used to compare the mean differences in GSE30122. Spearman correlation was used to determine the relationship between hub genes expressions and eGFR (Nephroseq V5). A *P*-value less than 0.05 was statistically significant. Receiver operating characteristic (ROC) curve analysis was performed using the ‘pROC’ package of R software to determine the sensitivity and specificity of target genes. Results were quantified by the area under the ROC curve (AUC), and genes with AUC > 0.85 were considered diagnostic. The ‘ggplot2’ package was used to draw boxplots and ROC curves.

### Ethics and consent

This study was approved by the medical ethics committee of Affiliated Hospital of Qingdao University *(Approval Number. QYFY-WZLL-27726)*. All microarray datasets were downloaded from the GEO database, and we confirmed that all necessary ethical approvals for animal experimentation and consent were obtained.

## Results

### Preprocessing analysis and identification of DEGs

**Figure 2:**
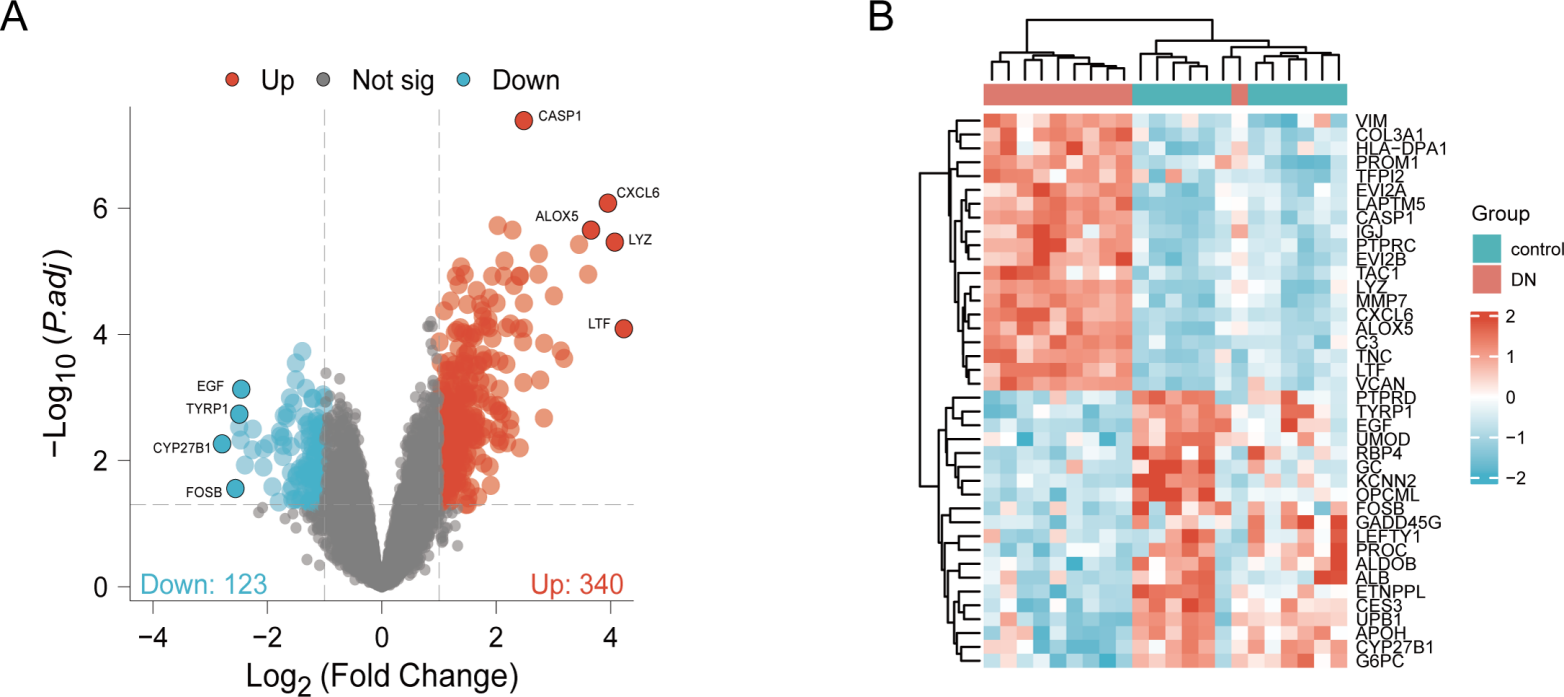
Identification of DEGs. (A) DEGs Volcano plot between the control and DN samples. The red dots represent up-regulated genes, the gray dots represent non-significant genes, and the blue dots represent down-regulated genes. (B) Heat-map of top 40 DEGs between the control and DN samples. Red rectangles represent up-regulated expression, and blue rectangles represent down-regulated expression.

DN and control samples from the GSE30529- GPL571 datasets consisted of 10 DN kidney tubules and 12 control samples. After GSE30529 was normalized, the box plot showed that samples had good distribution (Fig. S1A). We performed PCA to evaluate the repeatability of the expression matrix within GSE30529, and the results showed satisfactory repeatability based on the high proportion of variance explained by the first two principal components (PC1 and PC2) and the tight clustering of replicates in the PCA plot (Fig.S1B). Initially, we screened 19,820 genes in the microarray dataset GSE30529 after removing the duplicate probe. After setting criteria with the threshold of an adjusted absolute value of log2 (FC)> 1 and adj. *P* value < 0.05, 463 DEGs (340 increased and 123 reduced) were identified as DEGs. The DEGs are shown in Fig. 2A in volcano plots, and the top40 DEGs with the most distinct expression differences between DN and control samples are shown on the heat map (Fig. 2B).

### Enrichment analysis

**Figure 3:**
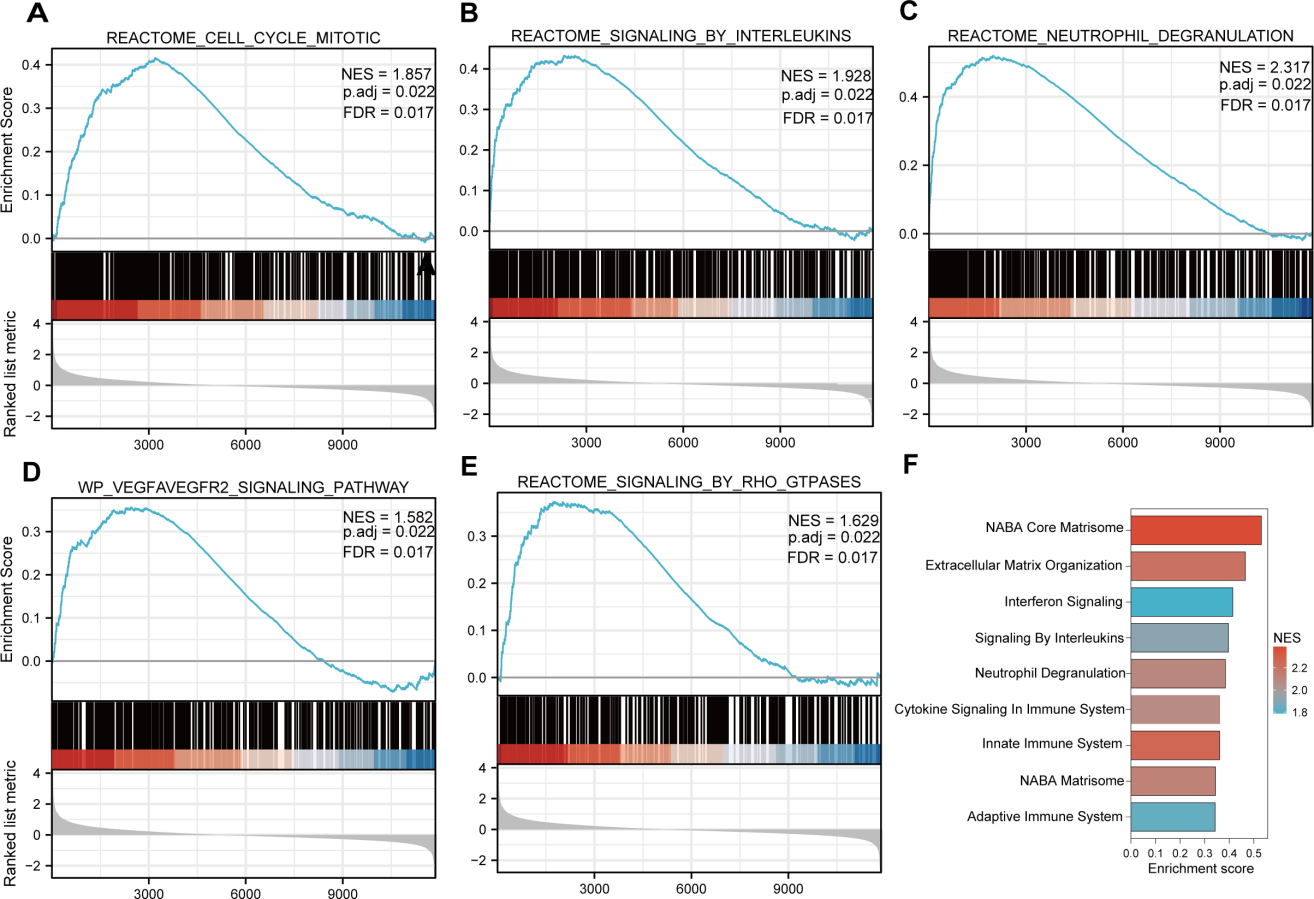
GSEA plots present most enriched gene sets between the control and DN groups. The C2 sub-collection CP gene collection was applied to conduct the canonical pathway enrichment analysis of the expression profile as a whole. (A–E) The top five most significant enriched gene sets are shown. (F) The bar plot shows the top enriched gene pathways. NES, normalized enrichment score; FDR, false discovery rates or adjusted *p*-value; ES, enrichment score.

**Figure 4:**
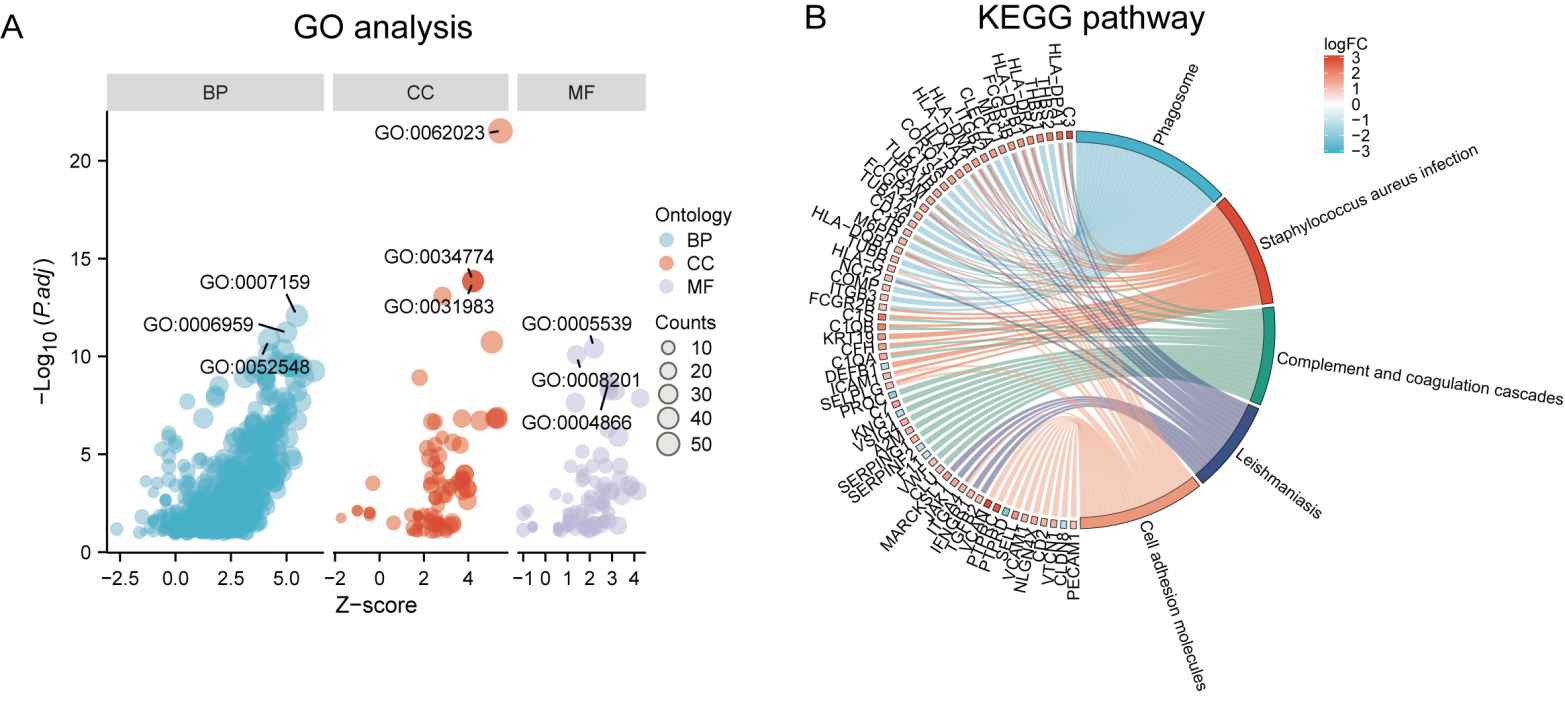
GO, KEGG pathway, and Reactome enrichment analyzes of DEGs. (A) The GO function analysis bubble chart shows the top three GO terms under each ontology including biological process (BP), cellular component (CC), and molecular function (MF). Bubble size indicates the counts contained in each term. (B) KEGG chord of the top five DEG-related pathways. The DEGs involved in the KEGG pathways are shown on the left.

Functional and pathway enrichment analyzes were performed by the R software ‘clusterProfiler’ package. First, the expression profiles of all genes in the DN and control samples were cleared and uploaded to GSEA, and the C2 sub-collection CP gene collection was applied to conduct the GO enrichment analysis. Adj. *p* < 0.05 and *Q* < 0.25 were set as the filtering criterion for significant gene sets. The most enriched gene sets were related to mitosis (Fig. 3A; normalized enrichment score (NES) = 1.857, false discovery rate (FDR) = 0.017), immune responses (Figs. 3B–3C; NES = 1.928, FDR = 0.017; NES = 2.317, FDR = 0.017), angiogenesis (Fig. 3D; NES = 1.582, FDR = 0.017), and cytoskeletal dynamics and morphology pathways (NES = 1.629, FDR = 0.017), as determined by the GSEA results. At the same time, the gene sets related to immune responses were found to be among the top-ranked pathways in terms of enrichment score, indicating their potential importance in the context of diabetic nephropathy (Fig. 3F). Next, the results of GO, KEGG pathway, GSEA, and Reactome enrichment analyses suggested that the immune response, including the regulation of neutrophil activation, response to interferon-gamma, neutrophil mediated immunity, and neutrophil degranulation, in DN samples was more prominent than in the control samples (Fig. 4A). KEGG enrichment analysis revealed a strong association between the identified DEGs and pathways related to neutrophil functions, such as phagosome (KEGG ID: hsa04145, adjusted *p*-value < 0.01), complement and coagulation cascades (KEGG ID: hsa04610, adjusted *p*-value < 0.01), and cell adhesion molecules (KEGG ID: hsa04514, adjusted *p*-value < 0.01) (Fig. 4B). More details of the enriched GO/KEGG pathway are included in Table 1. In addition, GO and KEGG enrichment analyses of revealed that immune-related biological processes and pathways were predominantly enriched. When looking at an immune-related nephropathic disease like DN, it is crucial to understand its immune mechanisms.

**Table 1:** GO and KEGG pathways.

**Ontology**	**ID**	**Description**	**p.adjust**
BP	GO:0042119	neutrophil activation	2.48E−15
BP	GO:0034341	response to interferon-gamma	1.36E−14
BP	GO:0002446	neutrophil mediated immunity	2.42E−14
BP	GO:0043312	neutrophil degranulation	2.90E−14
BP	GO:0002283	neutrophil activation involved in immune response	2.97E−14
CC	GO:0062023	collagen-containing extracellular matrix	2.30E−24
CC	GO:0034774	secretory granule lumen	1.41E−14
CC	GO:0060205	cytoplasmic vesicle lumen	4.40E−14
CC	GO:0031983	vesicle lumen	4.40E−14
CC	GO:0031091	platelet alpha granule	7.82E−14
MF	GO:0005539	glycosaminoglycan binding	9.45E−13
MF	GO:0008201	heparin binding	2.86E−12
MF	GO:1901681	sulfur compound binding	8.54E−10
MF	GO:0004866	endopeptidase inhibitor activity	8.95E−09
MF	GO:0030414	peptidase inhibitor activity	1.33E−08
KEGG	hsa04145	Phagosome	1.34E−12
KEGG	hsa05150	Staphylococcus aureus infection	2.83E−10
KEGG	hsa04610	Complement and coagulation cascades	1.33E−08
KEGG	hsa05140	Leishmaniasis	1.63E−08
KEGG	hsa04514	Cell adhesion molecules	1.08E−07
KEGG	hsa05152	Tuberculosis	1.34E−07
KEGG	hsa05323	Rheumatoid arthritis	1.40E−06
KEGG	hsa05144	Malaria	1.40E−06

**Notes.**

Annotation BP Biological Process CC Cellular Component MF Molecular Function

### PPI network analysis, MCODE cluster modules and hub gene identification

**Figure 5:**
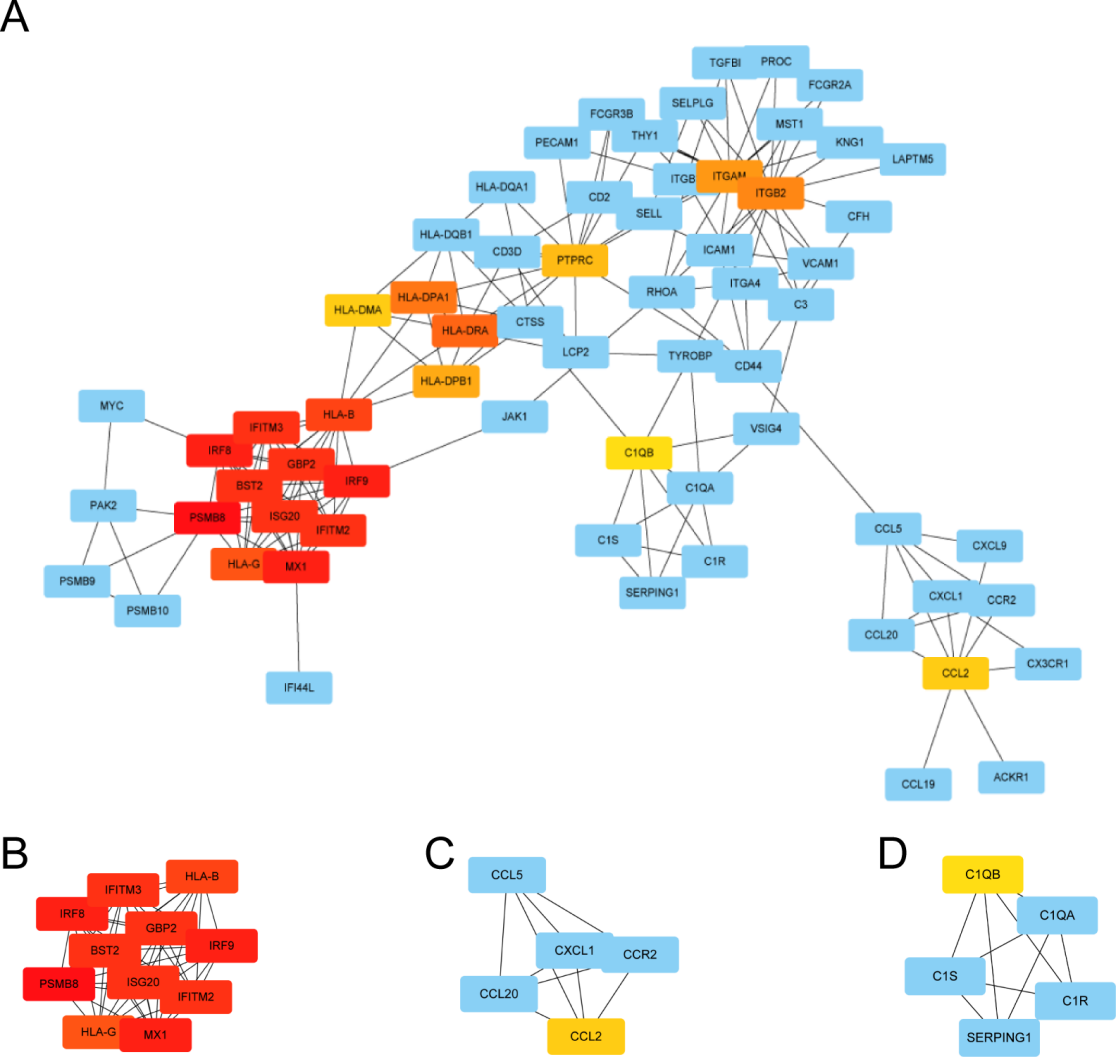
The protein–protein interaction network of DEGs and three cluster modules selected by MCODE. (A) The PPI network consists of 65 nodes and 183 edges. Protein represented by node and protein–protein association represented by edge. Non-blue nodes are the 20 hub genes screened by cytoHubba plug-in. MCODE extracted three cluster modules. Cluster 1 (B) has the highest cluster score (score: 10.8, 11 nodes and 54 edges), followed by cluster 2 (C) (score: 5, five nodes and 10 edges), and then cluster 3 (D) (score: 5, five nodes and 10 edges).

We used STRING to construct the interaction network between proteins coded by DEGs, which consisted of 65 nodes and 183 edges, and the network was visualized by Cytoscape (Fig. 5A). We used the cytoHubba plugin to identify hub genes. The MCC algorithms identified 20 hub genes. Three gene cluster modules were identified with the MCODE plugin (Figs. 5B–5D), according to the filter criteria. Details of these twenty hub genes identified by cytoHubba are given in Table 2. These genes, including PSMB8, IRF8, IRF9, MX1, BST2, GBP2, IFITM2, ISG20, IFITM3, HLA-B, HLA-G, HLA-DRA, HLA-DPA1, ITGB2, ITGAM, HLA-DPB1, PTPRC, CCL2, HLA-DMA, and C1QB, were the most crucial in the PPI network and played an important role in the pathogenesis of DN.

**Table 2:** Twenty hub genes identifed by cytoHubba.

**Gene symbol**	**Description**	**log2FC**	**Q value**	**Regulation**
PSMB8	Proteasome subunit beta type-8	1.342044794	0.000164732	UP
IRF8	Interferon regulatory factor 8	1.543495504	0.00054193	UP
IRF9	Interferon regulatory factor 9	1.015989833	0.000995947	UP
MX1	Interferon-induced GTP-binding protein Mx1	1.194190613	0.004106856	UP
BST2	Bone marrow stromal antigen 2	1.16619317	0.037461717	UP
GBP2	Guanylate-binding protein 2	1.447589391	0.003980602	UP
IFITM2	Interferon-induced transmembrane protein 2	1.216171959	0.020762203	UP
ISG20	Interferon-stimulated gene 20 kDa protein	1.018021414	0.00758014	UP
IFITM3	Interferon-induced transmembrane protein 3	1.241954263	0.020591992	UP
HLA-B	HLA class I histocompatibility antigen, B-7 alpha chain	1.499097739	0.000194636	UP
HLA-G	HLA class I histocompatibility antigen, alpha chain G	1.118671026	0.000373508	UP
HLA-DRA	Major histocompatibility complex, class ii, dr alpha	1.777805059	0.000258662	UP
HLA-DPA1	Major histocompatibility complex, class ii, dp alpha 1	2.468768075	0.000131723	UP
ITGB2	Integrin beta-2	1.538936861	0.000113105	UP
ITGAM	Integrin alpha-M	1.308463099	0.0000864	UP
HLA-DPB1	HLA class II histocompatibility antigen, DP beta 1 chain	1.727551628	0.00104215	UP
PTPRC	Receptor-type tyrosine-protein phosphatase C	2.737544685	0.0000112	UP
CCL2	C-C motif chemokine 2	1.772954596	0.000270229	UP
HLA-DMA	Major histocompatibility complex, class ii, dm alpha	1.526072847	0.000553678	UP
C1QB	Complement C1q subcomponent subunit B	2.021596465	0.001339804	UP

**Notes.**

Annotation FC fold change Q value adjust *P*-value

### Verification of the 20 hub genes by the GSE30122 database

**Figure 6:**
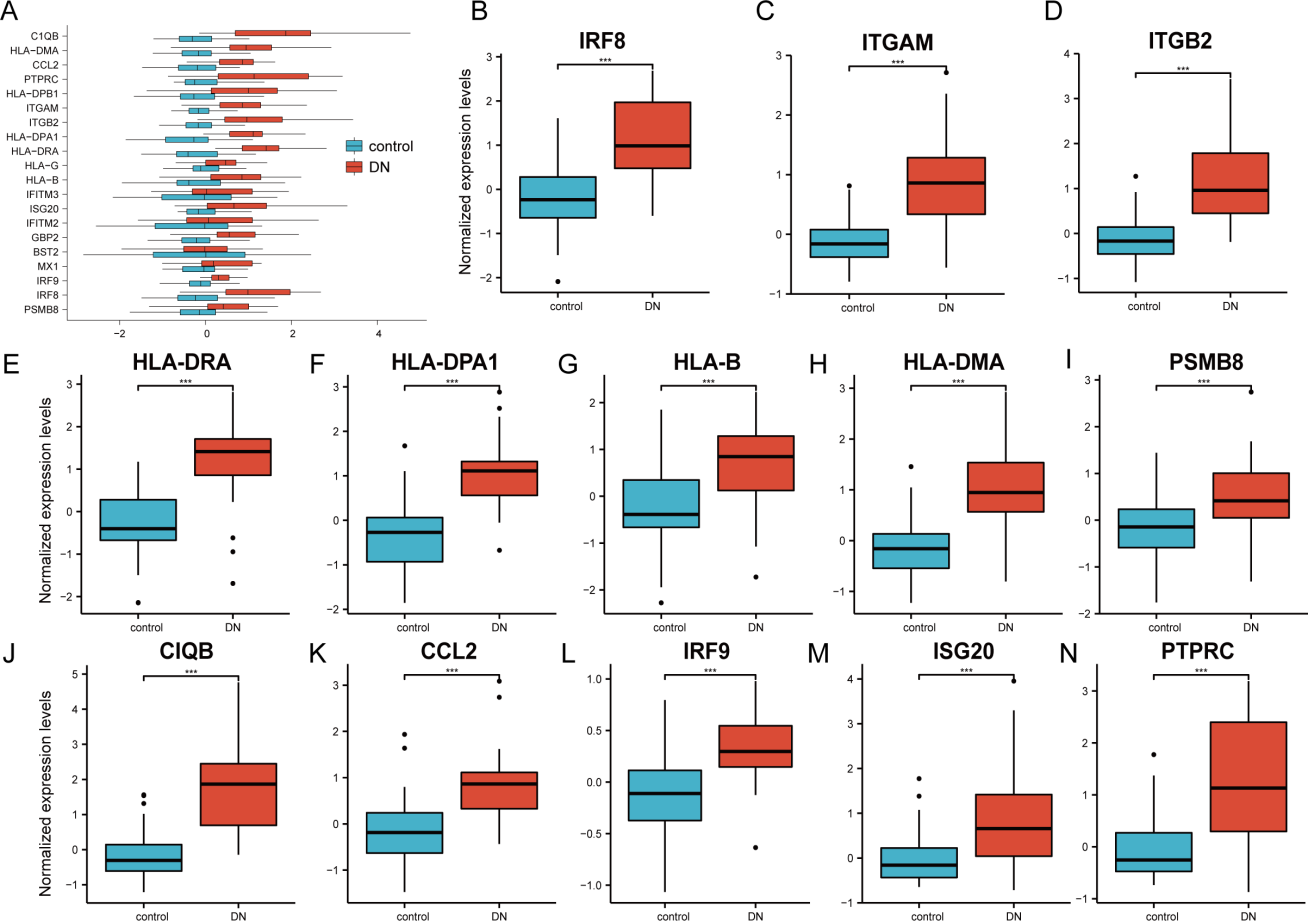
Verification of the 20 hub genes by the GEO database GSE30122. (A) Expression of 20 hub-genes in GSE30122 dataset between control and DN groups. (B–N) The 13 genes with the most significant statistical differences. ***: *p* < 0.001; **: *p* < 0.01; *: *p* < 0.05; ns: no significant difference.

The 20 hub genes were verified using the GPL17586- GSE30122 dataset, which consisted of 41 DN samples and 20 control samples. We used the R software ‘ggplot2’ package and ‘ggpubr’ package to construct box plots and perform Mann–Whitney U test statistical analysis. In line with our predictions, we found that the expression levels of the 20 hub genes in the DN group were different (Table 3), except for BST2 (Mann–Whitney U test, *p* = 0.753), IFITM2 (Mann–Whitney U test, *p* = 0.145), IFITM3 (Mann–Whitney U test, *p* = 0.161), and MX1 (Mann–Whitney U test, *p* = 0.054), which were higher than those in the control group (Fig. 6A). The expression levels of the hub genes in the control samples were decreased compared with those in the DN samples (Figs. 6B–6N).

**Table 3:** Statistical analysis of Hub-gene between control and DN groups. The *t test* was used when the observed variables were close to normal distribution in each group. When samples did not meet the requirements of normality test, Mann-Whitney U test (Wilcoxon Rank Sum test) was used.

**GENE**	**t/T**	Δ	**95% CI**	***p***
BST2	0.316	0.118	−0.627–0.863	0.753
C1QB	80	1.98	1.263–2.555	0.000
CCL2	5.128	1.075	0.657–1.494	0.000
GBP2	3.746	0.966	0.43–1.503	0.001
HLA-B	3.507	0.807	0.348–1.266	0.000
HLA-DMA	6.062	1.135	0.761–1.509	0.000
HLA-DPA1	6.375	1.408	0.967–1.849	0.000
HLA-DPB1	3.605	1.052	0.449–1.654	0.001
HLA-DRA	160	1.645	1.012–1.97	0.000
HLA-G	3.23	0.406	0.155–0.656	0.002
IFITM2	366	0.485	−0.203–1.174	0.145
IFITM3	1.418	0.388	−0.158–0.935	0.161
ISG20	211	0.686	0.324–1.175	0.000
IRF8	6.141	1.301	0.878–1.724	0.000
IRF9	4.487	0.462	0.257–0.668	0.000
ITGAM	4.841	1.038	0.592–1.485	0.000
ITGB2	5.155	1.296	0.773–1.819	0.000
MX1	331	0.459	−0.008–0.866	0.054
PSMB8	3.665	0.727	0.331–1.124	0.000
PTPRC	159	1.272	0.671–1.969	0.000

**Notes.**

The *t test* was used when the observed variables were close to normal distribution in each group. When samples did not meet the requirements of normality test, *Mann–Whitney U test* (Wilcoxon Rank Sum test) was used.

### The ROC curves of the 20 hub genes in DN and control groups

**Figure 7:**
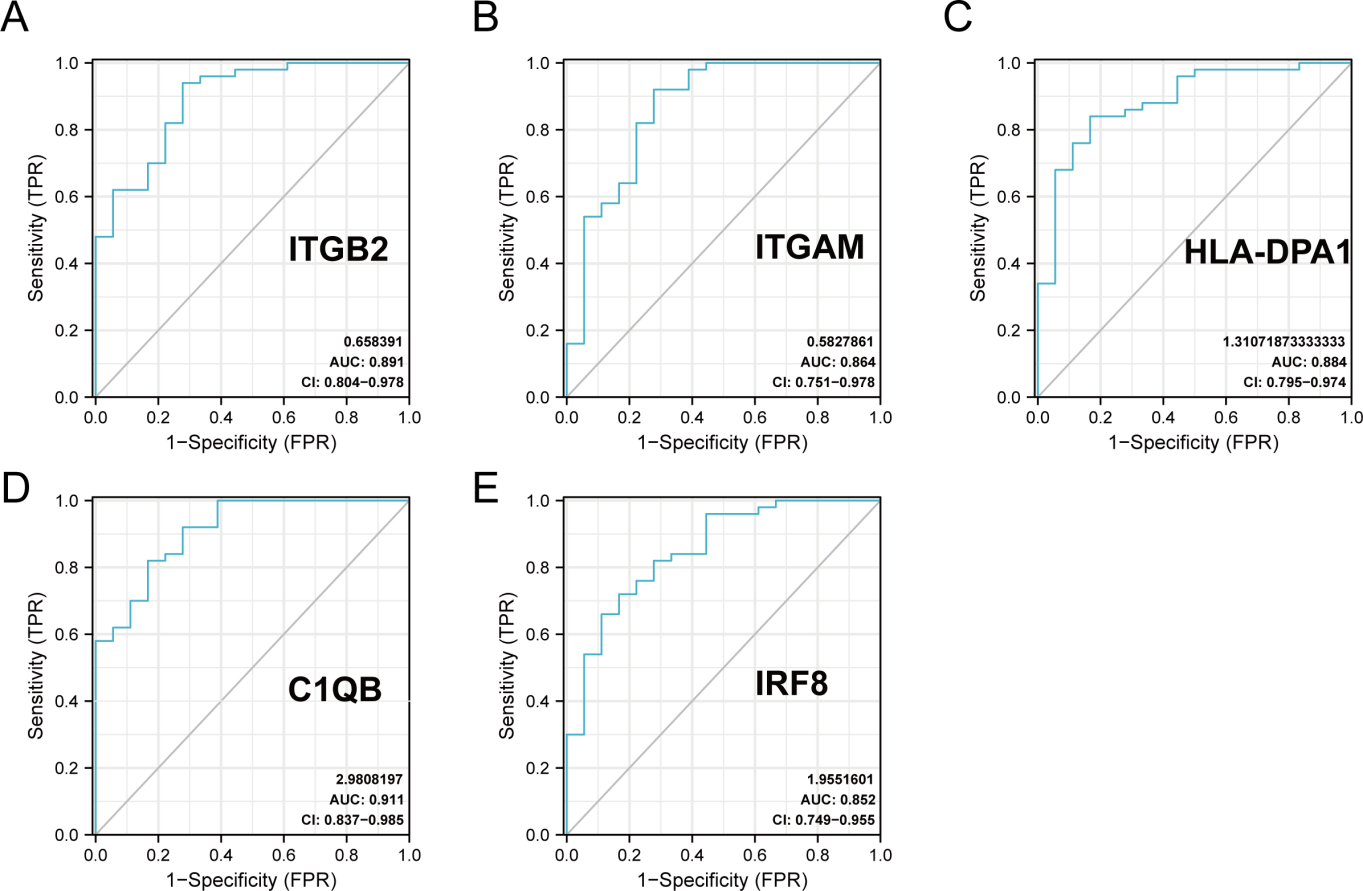
ROC curve of the hub genes from the GEO database GSE30122. (A–E) ROC curve of the hub genes with AUC > 0.85 in DN samples. CI, confidence interval; AUC, area under the ROC curve.

The R package ‘pROC’ was used to analyze the 20 hub gene expression profiles between the DN and control groups and paint the ROC curves. The AUC helped to evaluate sensitivity and specificity, and describe the original effectiveness of the diagnostic tests. Based on the statistical results, most of these hub genes had noteworthy diagnostic values in the DN group. Of these, CIQB had the largest AUC (AUC: 0.911). The ROC curves of genes with an AUC greater than 0.85 are shown in Fig. 7. We screened hub genes with AUC>0.85 to identify better candidate genes based on their good diagnostic performance in the DN group. C1QB, ITGB2, HLA-DPA1, ITGAM, and IRF8 levels were upregulated in DN with statistical significance after filtering, as determined by a *p*-value < 0.05 for upregulated genes. The specific *p*-values for each gene were: C1QB (Mann–Whitney U test, *p* < 0.01), ITGB2 (Mann–Whitney U test, *p* < 0.01), HLA-DPA1 (unpaired *t*-test, *t* = 6.375, *p* < 0.01), ITGAM (unpaired *t*-test, *t* = 4.841, *p* < 0.01), and IRF8 (unpaired *t*-test, *t* = 6.141, *p* < 0.01) (Table 4). Accordingly, we speculate that C1QB, ITGB2, HLA-DPA1, ITGAM, and IRF8 could be potential candidate genes for DN diagnosis based on our current results.

**Table 4:** The evaluation of the hub-gene as prognostic predictor.

**gene**	**AUC**	**95% CI**	**Optimal cut-off value**	**sensitivity**	**specificity**
BST2	0.508	0.360–0.655	−0.801	0.38	0.778
C1QB	0.911	0.837–0.985	0.244	0.82	0.833
CCL2	0.838	0.731–0.945	0.163	0.72	0.833
GBP2	0.829	0.686–0.971	0.292	0.92	0.722
HLA-B	0.761	0.614–0.909	0.379	0.78	0.722
HLA-DMA	0.846	0.719–0.972	0.449	0.84	0.833
HLA-DPA1	0.884	0.795–0.974	0.388	0.84	0.833
HLA-DPB1	0.781	0.639–0.923	0.323	0.84	0.667
HLA-DRA	0.823	0.674–0.973	1.175	0.98	0.722
HLA-G	0.749	0.604–0.894	0.424	0.88	0.611
IFITM2	0.608	0.449–0.767	0.962	0.92	0.333
IFITM3	0.587	0.432–0.741	−1.27	0.22	1.000
ISG20	0.771	0.631–0.912	0.645	0.94	0.556
IRF8	0.852	0.749–0.955	0.149	0.72	0.833
IRF9	0.818	0.697–0.938	0.012	0.66	0.889
ITGAM	0.864	0.751–0.978	0.496	0.92	0.722
ITGB2	0.891	0.804–0.978	0.719	0.94	0.722
MX1	0.636	0.477–0.795	0.54	0.84	0.444
PSMB8	0.749	0.604–0.894	0.206	0.74	0.722
PTPRC	0.827	0.696–0.957	0.54	0.86	0.722

**Notes.**

AUC area under the ROC curve CI confidence interval

### The co-regulatory network of miRNA-mRNA-TF

**Figure 8:**
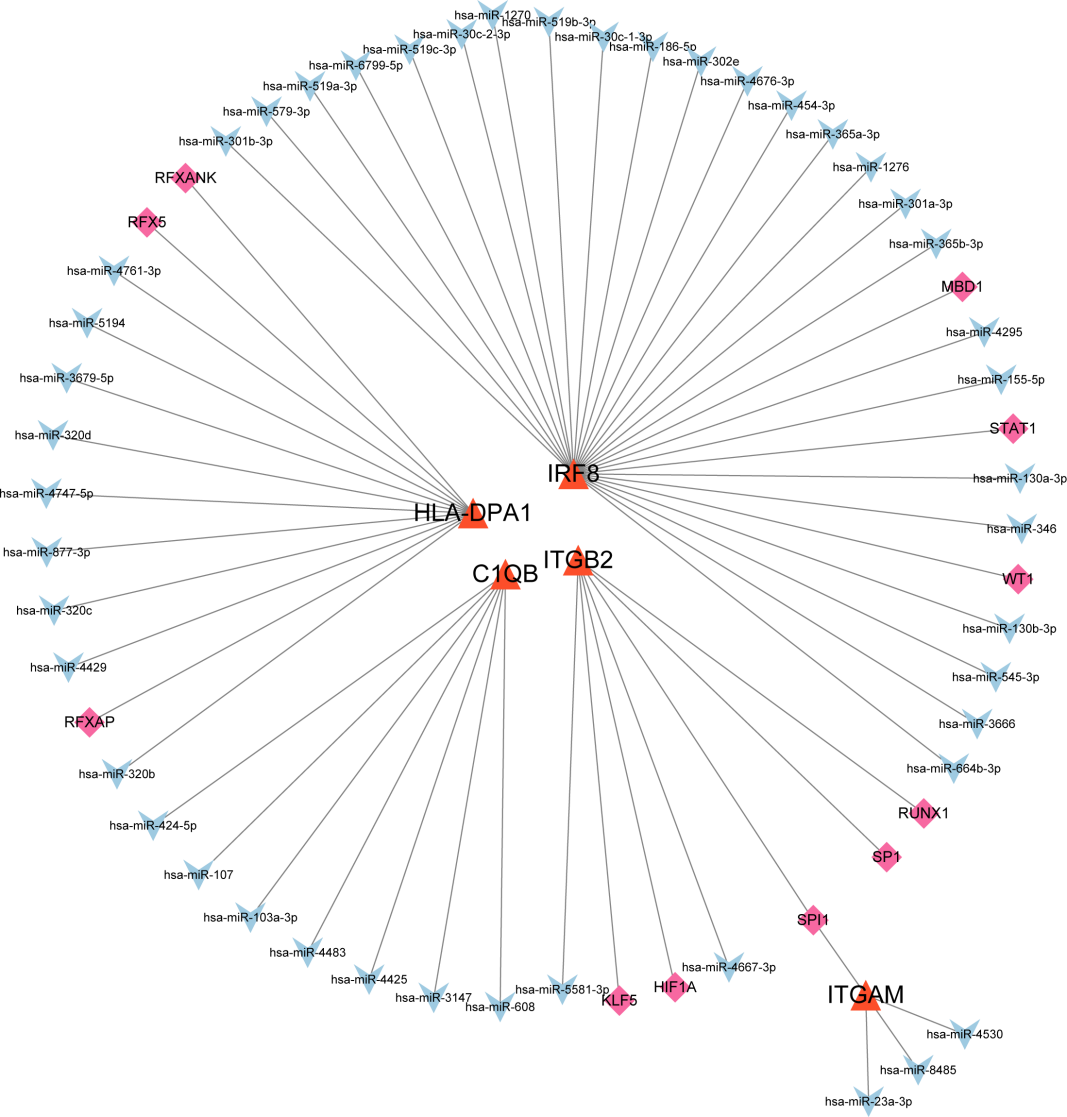
MiRNA–mRNA–TF regulatory network of five selected hub genes. The green diamonds represent TFs, the blue Vs represent MiRNAs, and the orange triangles represent selected hub genes.

After searching the databases (miRWalk3.0, TargetScan, MiRDB, and starBase) for scores > 0.95, we identified 46 miRNAs acting on a 3′ UTR region of the genes as potential biological prognostic predictor. Subsequently, 11 TFs (HIF1A, KLF5, MBD1, RFX5, RFXANK, RFXAP, RUNX1, SP1, SPI1, STAT1, and WT1) were screened using the online database TRRUST (Table 5). Finally, using the results of the screening, we predicted 46 miRNA–mRNA pairs and 13 TF-mRNA pairs and then integrated the predicted pairs to describe a TF–miRNA–mRNA regulatory network constructed using Cytoscape (Fig. 8). Among these regulatory networks, we noted that IRF8 had the highest number of miRNA connections, which formed 25 miRNA-mRNA pairs in total. TFs with the highest concentration were found on ITGB2 including HIF1A, KLF5, RUNX1, SP1, and SPI1. It is noteworthy that of the five genes, only C1QB had no associated TFs based on the predicted outcomes.

**Table 5:** Target gene co-regulatory network.

Target gene	TF	miRNA
ITGB2	none	hsa-mir-26b-5p hsa-mir-146a-5p hsa-mir-335-5p hsa-mir-148b-5p hsa-mir-17-3p hsa-mir-22-5p hsa-mir-3677-5p hsa-mir-182-5p hsa-mir-27a-3p
ITGAM	ARID1B POLR2A IRF1	hsa-mir-191-5p hsa-mir-26a-5p hsa-mir-372-3p
IRF8	ZNF143 NFIC TCF7 ZNF76 WRNIP1 MTA2 EBF1 CBFB TBX21 EED EZH2 POU2F2	hsa-mir-130a-3p hsa-mir-130b-3p hsa-mir-146a-3p hsa-mir-181a-5p hsa-mir-181c-5p hsa-mir-186-5p hsa-mir-218-5p hsa-mir-3605-3p hsa-mir-365a-3p hsa-mir-365b-3p hsa-mir-664a-3p hsa-mir-101-3p hsa-mir-146a-5p
HLA-DPA1	ATF1 WRNIP1 RFXANK HDGF CBFB ZNF382 STAT1 MAZ RFX5 PML CEBPB	hsa-mir-1343-3p hsa-let-7a-5p hsa-let-7b-5p hsa-let-7f-5p hsa-let-7g-5p hsa-let-7i-5p hsa-mir-1179 hsa-mir-146a-5p hsa-mir-146b-5p hsa-mir-148a-5p hsa-mir-148b-5p hsa-mir-21-3p hsa-mir-320a hsa-mir-320b hsa-mir-320c hsa-mir-320d hsa-mir-342-5p hsa-mir-3679-5p hsa-mir-374a-5p hsa-mir-374b-5p hsa-mir-589-5p hsa-mir-7-5p hsa-mir-98-5p hsa-mir-126-3p hsa-mir-129-2-3p hsa-mir-200c-3p hsa-mir-214-3p hsa-mir-26a-5p
C1QB	NR2F1 YBX1 ADNP	hsa-mir-26b-5p hsa-mir-124-3p hsa-mir-129-2-3p hsa-mir-146a-5p

**Notes.**

TF Transcription factors miRNA MicroRNA

### Hub gene expression was positively correlated with the degree of kidney injury

**Figure 9:**
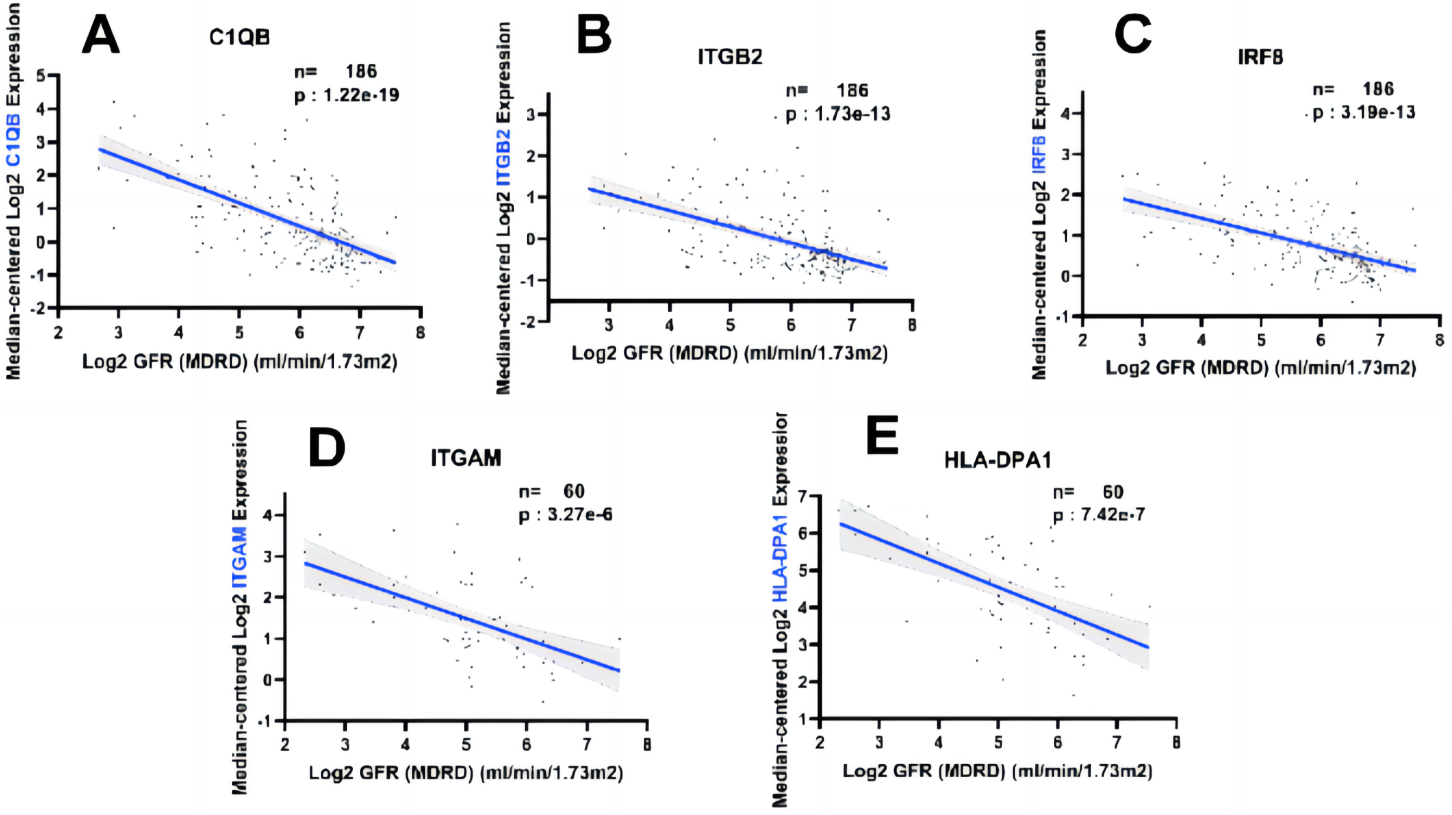
Relationship between five hub genes as biological predictors and renal function. Glomerular filtration rate (GFR) was assessed by modification of diet in renal disease (MDRD) equation.

In order to fully explore the clinical value of the selected five hub genes, we screened two gene expression datasets containing 186 and 60 CKD patients, respectively (Saint-Mezard et al., 2009; Ju et al., 2015). Correlation analysis between biological predictors and GFR was carried out in the Nephroseq V5 database (Figs. 9A–9E). All five biological predictors were negatively correlated with GFR, with the following Pearson correlation coefficients and *p*-values: C1QB (r =−0.601, *p*-value < 0.01), ITGB2 (r =−0.506, *p*-value < 0.01), HLA-DPA1 (r = −0.589, *p*-value < 0.01), ITGAM (r = −0.560, *p*-value < 0.01), and IRF8 (r =−0.501, *p*-value < 0.01). Thus, a higher expression of those hub genes indicated worse renal function in patients with CKD, which may play a role in kidney deterioration and damage in patients with CKD.

### Validation of five hub genes’ expression with qRT-PCR

**Figure 10:**
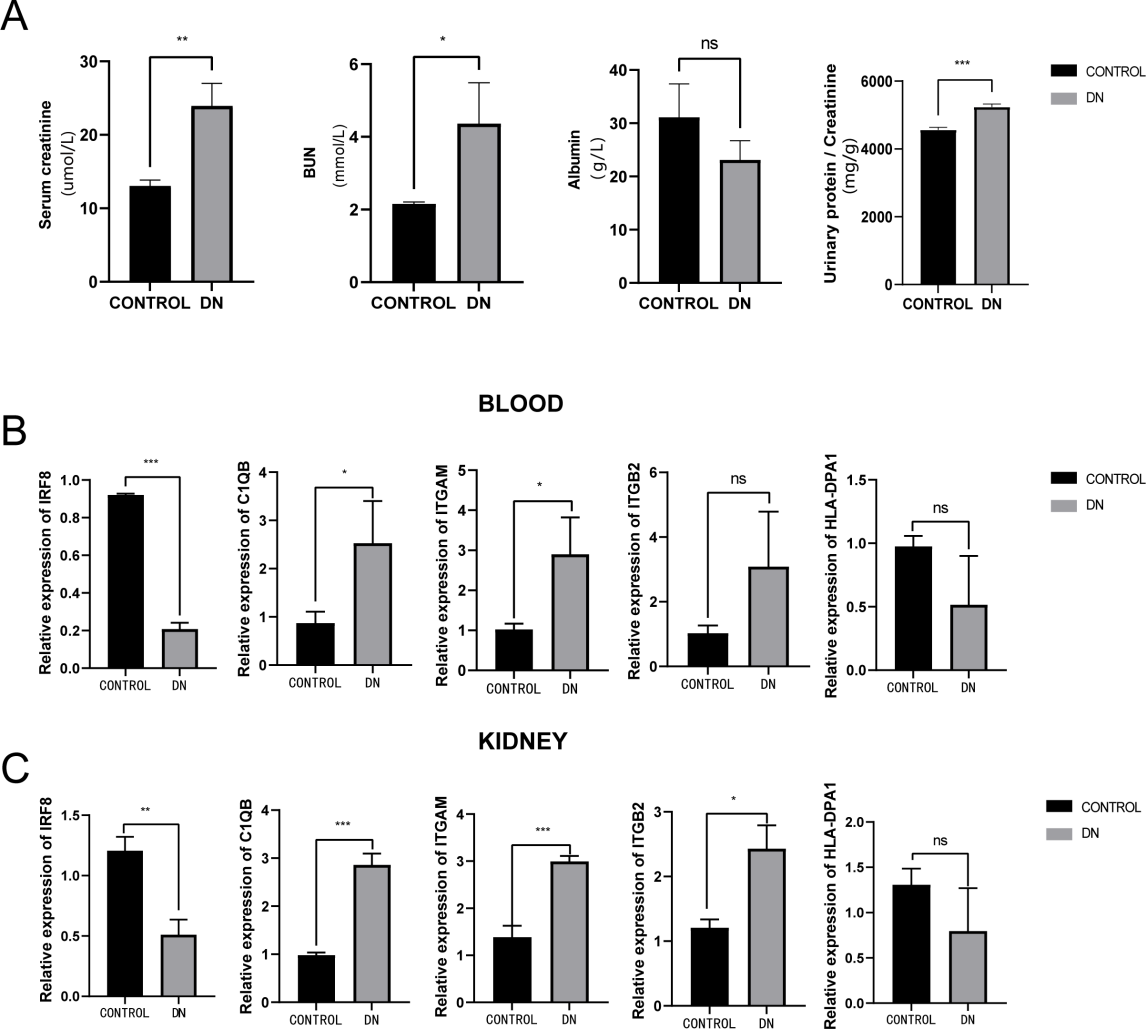
Evaluation of a diabetic renal rat model and RT-qPCR analysis of five Hub genes. (A) The serum level of creatinine, BUN, albumin and urinary protein/creatinine ratio. (B) Validation of the expression level of hub genes in blood samples. (C) Validation of the expression level of hub genes in kidney samples. ***: *p* < 0.001; **: *p* < 0.01; *: *p* < 0.05; ns, no significant difference; BUN, blood urea nitrogen; DN, diabetic nephropathy.

To further assess the expression of these hub genes, a total of three DN model rats and three healthy controls were enrolled as a validation cohort. We first measured serum creatinine, BUN, and albumin levels, and calculated the urinary protein/creatinine ratio in the DN rat group and control group. We found that the level of kidney injury markers, such as serum creatinine and BUN, in the DN group was higher than in the control group (unpaired *t*-test, *t* = 3.391, *df* = 4, *p* = 0.0275, *r* = 0.861). Meanwhile, the DN group had a higher urinary protein/creatinine ratio (unpaired *t*-test, *t* = 17.23, *df* = 16, *p* < 0.001, *r* = 0.974), suggesting more significant proteinuria (Fig. 10A). RT-PCR technology was then used to confirm the differential expression levels from participant serum and kidney samples. Consistent with the microarray data, ITGAM (unpaired *t*-test, *t* = 10.35, *df* = 4, *p* < 0.001, *r* = 0.981) and C1QB (unpaired *t*-test, *t* = 13.60, *df* = 4, *p* < 0.001, *r* = 0.989) expression were significantly upregulated in both serum and kidney samples (Figs. 10B and 10C) between DN and control groups. At the same time, ITGB2 was highly expressed in the kidney samples in the disease group (unpaired *t*-test, *t* = 5.512, *df* = 4, *p* = 0.0053, *r* = 0.940). However, there was no difference in the levels of HLA-DPA1. Notably, the expression level of IRF8 in the DN group was lower than in the control group, which was contrary to our prediction.

## Discussion

DN is usually diagnosed on the basis of increased urine albumin-creatinine ratio (UACR) or decreased estimated glomerular filtration rate (eGFR) when a patient has no other CKD. However, DN diagnosis relies heavily on assumptions and updated specific DN markers are urgently needed, especially for early-stage disease (Forbes & Cooper, 2013; Ioannou, 2017). Therefore, in this study, based on the GSE30529 microarray data taken from the GEO database, we screened DEGs between the DN and control group and identified 463 DEGs (340 increased genes and 123 decreased genes).

According to our GSEA, the expression levels of the immune inflammatory response related signaling pathways, such as the interleukin and neutrophil degranulation pathways were upregulated in the DN group. This may reveal the cause of glomerular vascular injury, glomerulosclerosis, nodular lesions, and deterioration of renal function. The GO analysis concluded that DEGs were involved in neutrophil activation, neutrophil-mediated immunity, neutrophil degranulation, neutrophil activation, and response to interferon-gamma, which indicates that the level of immune response and regulation in DN group was more obvious than that in the control. In addition, the expression of DEGs may act on the molecular functions of glycosaminoglycan binding, heparin binding, sulfur compound binding, endopeptidase inhibitor activity, and peptidase inhibitor activity, indirectly demonstrating the complexity of the DN’s pathogenesis. The enriched pathways identified by KEGG analysis included phagosome, staphylococcus aureus infection, complement and coagulation cascades, leishmaniasis, cell adhesion molecules, tuberculosis, rheumatoid arthritis, and malaria. Thus, based on GSEA, GO, and KEGG enrichment analysis results, DN showed strong immune activation and signal transduction, which is in agreement with previous studies (Wada & Makino, 2013).

We used the GSE30122 microarray dataset, including 19 DN and 50 control samples for verification after constructing a PPI network to identify 20 hub genes. Statistical analysis of hub gene expression levels explained that the expression level of most hub genes in DN was higher than in the control group, and there was a statistically significant difference (*P* < 0.05). In the ROC curve analysis, the AUC of C1QB, HLA-DPA1, IRF8, ITGAM, and ITGB genes were higher than 0.85, indicating a higher DN predictive value. Therefore, based on the five genes screened above, we constructed a miRNA-mRNA-TF regulatory network using the online database to try to interpret the pathogenesis of DN in the process of gene expression. We then evaluated the association of selected genes with kidney injury using the Nephroseq V5 database. At the same time, we further verified the expression levels in both serum and kidney samples of these five genes using the qPCR test and a DN rat model, and the results confirmed our previous prediction.

Complement C1q B chain (C1QB) is a protein coding gene. C1q associates with the proenzymes C1r and C1s to yield C1, the first component of the serum complement system (Reid, 2018). In the blood plasma, the complement system is a proteolytic cascade that mediates innate immunity, a non-specific defense mechanism to pathogens (Daugan et al., 2017). The main consequences of complement activation are the opsonization of pathogens, recruitment of inflammatory and immunocompetent cells, and direct killing of pathogens. Although complement cascades are an important part of the innate immune system, uncontrolled activation can lead to serious disease. One recent review found that excessive complement activation in atypical hemolytic uremic syndrome resulted in renal failure if not treated (Willows, Brown & Sheerin, 2020). Experimental and clinical evidence suggests that several components of the complement system are involved in the pathogenesis of DN (Flyvbjerg, 2017). The deposition of membrane attack complex formation (MAC) in the complement system is believed to be closely related to the pathogenesis of advanced DN (Fearn & Sheerin, 2015). Lu et al.’s (2022) study suggested that C1 and C2 could be two distinct immune-associated genes in the pathogenesis of DN. Sun et al. (2019) found an association between C1q and C3c complement deposition on renal histopathology and more severe kidney damage in DN patients. Our results provide evidence that C1QB is highly expressed in DN models and also emphasize the roles of C1 and local complement activation in DN, while the expression of C2 and C3 related genes is worthy of further investigation.

The ITGAM gene encodes the integrin alpha M chain, and ITGB2 encodes an integrin beta chain that combines with multiple different alpha chains to form different integrin heterodimers. Integrins are integral cell–surface proteins that participate in cell adhesion as well as cell–surface mediated signaling (Siegers, 2018). Integrin ITGAM/ITGB2 is a receptor for the iC3b fragment of the third complement component, which is involved in the composition of the complement system that enhances macrophage apoptotic neutrophil phagocytosis (Kristóf et al., 2013). ITGB2 was identified as a central gene in the complement cascade pathway that is negatively correlated with glomerular filtration rate (GFR; Xu et al., 2021). Complement activation contributes to MAC formation, which drives neutrophil activation and endothelial injury (Riedl et al., 2014). Meanwhile, integrin ITGAM/ITGB2 can recognize P1 and P2 peptides of fibrinogen gamma chain and is also a receptor for coagulation factor Xa (FXa). Fibrinogen is a soluble glycoprotein that plays an important role in the coagulation system (Mosesson, 2005) and inflammation (Davalos & Akassoglou, 2012). There is increasing evidence showing that serum fibrinogen, an acute phase marker of inflammation, plays an important role in regulating the inflammatory response and aggravating the progression of renal disease by binding to receptors expressed on the surface of different cells (Wang et al., 2017). Elevated plasma fibrinogen levels have been shown to be an indicator of a proinflammatory state and are closely associated with diabetes (Le et al., 2008). In addition, FX is mainly synthesized in the liver (Borensztajn, Peppelenbosch & Spek, 2008). However, there is evidence that FX extrahepatic expression by macrophages contributes to the development of asthma (Shinagawa et al., 2007). FXa activates PAR-1 and PAR-2 to enhance inflammation (Jose & Manuel, 2020). DM directly increased FX expression in macrophages (Oe et al., 2016). Local FX synthesis in macrophages infiltrating the kidney may contribute to DN progression. In our study, although these two genes were consistent as previously predicted and showed differences in expression between different groups of kidney tissue samples, we found that the level of differences in expression between groups of ITGAM was more significant and statistically maintained in blood samples. Previous studies did not examine the gene expression levels in specific samples in order to focus on differences in expression between the two (Hu et al., 2021). Our validation test results suggests that ITGAM can be a better predictor of DN than ITGB2. Blood samples are more accessible to obtain than kidney samples in clinical practice, which further increases the value of testing ITGAM to predict DN.

HLA-DPA1 is a member of the class II alpha chain HLA mimicry. Class II is a heterodimer consisting of an *α* chain (DPA) and *β* chain (DPB), both anchored to the cell membrane. It plays an important role in the immune system and manifests itself as peptides produced by extracellular proteins. Class II molecules are expressed in antigen presenting cells (APC) such as B lymphocytes, dendritic cells, and macrophages. Previous studies have shown that HLA-DPA1, the closest centromere gene expressed in HLA-DO *α*, may increase the risk of diabetes and diabetic kidney disease (DKD), which is a complication of diabetes (Varney et al., 2010). This gene also plays a role in the rheumatoid arthritis (RA) and systemic lupus erythematosus (SLE) related pathways (Ma et al., 2017). Consistent with Ma et al.’s (2017) research, the conclusion obtained from our preliminary analysis based on the microarray suggested that HLA-DPA1 was highly expressed in the disease group, while our q PCR verification results suggested that there was no statistical difference between the groups. These results show that further research is needed to determine whether HLA-DPA1 in DN can affect pathologic processes, similar to those in RA and SLE.

The interferon (IFN) regulatory factor (IRF) family specifically binds to the upstream regulatory region of type I IFN and IFN-inducible MHC class I genes (Salem et al., 2014). IRF family proteins bind to IFN-stimulated response elements (ISRE) and adjust the expression of genes, namely IFN-α and IFN-β. In diabetic mice, the expression of IRF8 is closely related to the activation of microglia and the regulation of the inflammatory response, thereby influencing the occurrence of diabetic retinopathy (Liu, Xu & Gao, 2021). However, the role of IRF8 in DN, which is also a microvascular complication of diabetes, still needs to be further investigated. As a TF, IRF8 has a complex regulatory relationship with other TFs. STAT1 binds to the IRF promoter region in colon cancer cells, while MBD1 inhibits IRF8 expression (McGough et al., 2008), which is another indication evidence that WT1 in leukemia and IRF8 are anticorrelated (Vidovic et al., 2010). Therefore, it is necessary to further explore the interaction between IRF8 and other transcription related factors in DN. In addition, the results from the online database indicated an inverse relationship between IRF8 and renal function, while qPCR indicated a low expression level of IRF8 in the DN group. These differences may be caused by the different regulatory effects of IRF8 as a TF in different species.

Notably, in the miRNA-mRNA-TF networks, all of the 11 predicted TFs have been proven to be key modulators and potential therapeutic targets for various inflammatory diseases. The TF SPI1 could regulate the expressions of both ITGAM and ITGB2.SPI1 encodes an ETS-domain TF that activates gene expression during myeloid and B-lymphoid cell development (Wittwer, Marti-Jaun & Hersberger, 2006). This was consistent with our analysis which showed that SPI1 expression was significantly increased in DN, suggesting that DN progression may be related to bone marrow cell activation and B-lymphoid cell development.

MicroRNA is one of the most important endogenous epigenetic factors that can inhibit the post-transcriptional gene expression of target genes. Recent studies have revealed that miRNAs may be regulators of immune and inflammatory responses and are potential therapeutic targets in DN (Kato & Natarajan, 2015; Zhou et al., 2021). Among the miRNAs predicted by the online databases, some have been shown to be closely related to the occurrence and development of diabetes. For example, hsa-miR-424-5p binds to PD-1 signaling molecules, stimulates the immune response through the mTORC signaling pathway, and is involved in the pathogenesis of type 1 diabetes (Wang et al., 2020). There is evidence that these miRNAs also act as protective factors in other diseases. Mir-5581-3p plays an inhibitory role in the progression of hepatocellular carcinoma (HCC) by regulating the expression of cardiolipin synthase 1 (CRLS1; Yin et al., 2019). The lower expression level of mir-8485 is associated with significantly lower overall survival rate of oral squamous cell carcinoma (OSCC; Gholizadeh et al., 2020). Mir-8485 can also alleviate cardiomyocyte injury in chronic heart failure (CHF) by targeting TP53INP1 (Luo et al., 2022). However, whether the predicted miRNAs can participate in regulating cell viability, apoptosis, inflammatory responses in DN, and disease progression still deserves further investigation.

Although this is the first study to construct a miRNA-mRNA-TF regulatory network to explore potential predictors of DN through two microarray datasets, this study still had several limitations. First, due to the small number of data sets integrated, only data sets related to DN genes were obtained from NCBI database, so other undiscovered DEGs still need to be explored based on multiple databases. Second, although identified hub genes have been reported to be closely related to the regulation of immune responses, there is still little evidence that they can clearly regulate immune responses in the DN disease process (Li et al., 2021). The observed high expression of immune-related genes in DN may be due to the enrichment of immune cells in DN tissue. Thus, more precise and detailed studies at the cell or animal level, such as single cell sequence studies, are needed to further validate the potential factors identified in our bioinformatics findings. Third, our qPCR results were based on rat models, but there are differences in gene expression between species. Lastly, we recognize that the number of laboratory animals used in our study was limited. The small sample size may have led to inconclusive results, and validation with larger cohorts of animals is needed to strengthen the evidence supporting our bioinformatics findings. Therefore, our prediction results need to be carried out across large clinical cohort studies or randomized controlled studies to evaluate its actual clinical value.

## Conclusion

In summary, we conducted comprehensive analysis and validation based on multiple microarray datasets, and the results showed that C1QB, ITGAM, and ITGB may be potential candidate genes that are upregulated in DN. These genes have remarkable diagnostic properties. The predicted miRNA-mRNA-TF pathway network was built based on online databases. Our results at the transcriptional level may operate as reference when exploring the pathogenesis of DN in the future. It is important that these interactions be treated with caution given the complexity of miRNA and TF crosstalk.

##  Supplemental Information

10.7717/peerj.15437/supp-1 Supplemental Information 1 Biochemica indexes The results of biochemical measurements on rat models.

10.7717/peerj.15437/supp-2 Supplemental Information 2 The level of group expression difference of the selected genes in GSE30122

10.7717/peerj.15437/supp-3 Supplemental Information 3 GSE30529 differential analysis The differential analysis results after normalized processing.

10.7717/peerj.15437/supp-4 Supplemental Information 4 DEGs selec A list of input genes for the String database

10.7717/peerj.15437/supp-5 Supplemental Information 5 PCR data The data results of validation of hub-genes using PCR.

10.7717/peerj.15437/supp-6 Supplemental Information 6 ARRIVE 2.0 Checklist

10.7717/peerj.15437/supp-7 Supplemental Information 7 (A) Box plot showing the distribution of normalized samples; (B) PCA plot of GSE30529 microarray Red and blue spots represent samples from DN and control group, namely.

10.7717/peerj.15437/supp-8 Supplemental Information 8 The genes under different screening conditions > 0.4 or > 0.7

10.7717/peerj.15437/supp-9 Supplemental Information 9 4 genes not in the intersection under the > 0.7 condition

10.7717/peerj.15437/supp-10 Supplemental Information 10 PCR melt curve analysis results

10.7717/peerj.15437/supp-11 Supplemental Information 11 Primer sequences for qPCR analysis

## Additional Information and Declarations

### Data Availability

The following information was supplied regarding data availability: The raw measurements are available in the Supplemental Files.
