# Supplementary material for: Identifying C1QB, ITGAM, and ITGB2 as potential diagnostic candidate genes for diabetic nephropathy using bioinformatics analysis
Source: PeerJ. 2023 May 25;11:e15437. doi: 10.7717/peerj.15437 (PMC10225123; doi:10.7717/peerj.15437)
Supplement: Supplemental Information 5 — The data results of validation of hub-genes using PCR. [file peerj-11-15437-s005.zip › kidney sample/PCRprotocol-kidney.pdf]

Report of the fluorescence quantitative PCR experiment

I、Experimental parameters

Subject: Rat kidney tissue

Experimental groups: normal group, diabetic group

Target genes: C1QB, ITGAM, ITGB2, HLA-DPA1, IRF8

II、Laboratory equipment and reagents

1. Laboratory equipment

The name of the	manufacturer	model
High speed cryogenic tissue grinder	Servicebio	KZ-III-F
Taiwan high speed freezer centrifuge	Hettichi, Germany	The 0004219-11
Fluorescence quantitative PCR instrument	bole	CFX connect
Ultra clean workbench	Suzhou purification	SW-CJ-1FD
Ultramicro spectrophotometer	Thermo Fisher	M mu Ltiskan sky
Pipetting gun	Eppendorf Research®plus	3120000348
Pure water meter	leacom	SMART-N
TIP # head	Shandong Baroque	20-1000.

2. Experimental reagents

reagent	manufacturer	number

Ultrapure RNA extraction kit	Kangwei Century	CW0581M
Trichloromethane	Sinopharm Group Chemical Reagent Co	10006818
TRIzon Reagent	Kangwei Century	CW0580S
Isopropyl alcohol	Sinopharm Group Chemical Reagent Co	80109218
Absolute ethanol	Sinopharm Group Chemical Reagent Co	10009218
SYBR Green Pro Taq HS Premixed qPCR Kit	Akoray Bio	AG11701
Evo M-MLV reverse transcription reagent premix	Akora Bio	AG11706
primer	Beijing Dingguo Bio	

The centrifuge tube and TIP head should be sterilized with damp heat for 20min and dried.

III. Experimental steps of fluorescence quantitative PCR

1. Total RNA extraction (the tip and centrifuge tube are sterilized by damp heat, without RNA enzyme)

(1) Animal tissues: Take fresh or -70°C frozen animal tissues and cut them into pieces as far as possible, add 1 mlTrizon Reagent every 30-50mg of tissue, and homogenize with a homogenizer.Or after grinding in liquid nitrogen, add 1ml TRIzon Reagent and mix.

Note: The sample volume should generally not exceed 20% of the volume of TRIzon Reagent.

(2) Suck TRIzon Reagent into a 2mL centrifuge tube, suck and mix well, and let stand for 5min.

(3) Add 200 µL chloroform per 1ml TRIzon Reagent, shake violently for 45 seconds to make it fully mixed, the liquid is emulsion, and let stand for 3min at room temperature.

(4) Centrifugation at 12000rpm for 10min at 4 ° C.

(5) Transfer the supernatant to a new centrifuge tube, add an equal volume of 70% ethanol, and mix upside down.
(6) Add all the solution obtained in the previous step to the adsorption column that has been loaded into the collection tube.If it can not be added at one time, it can be transferred several times.Centrifuge at 12000rpm for 20 seconds, pour out the waste liquid in the collection tube, and put the adsorption column back into the collection tube.
(7) Add 700μL Buffer RW1 to the adsorption column, centrifuge at 12000rpm for 20 seconds, pour out the waste liquid in the collection tube, and put the adsorption column back into the collection tube.
(8) Add 500μL Buffer RW2 to the adsorption column (check whether absolute ethanol has been added before use), centrifuge at 12000rpm for 20 seconds, pour out the waste liquid in the collection tube, and put the adsorption column back into the collection tube.
(9) Repeat step 8.
(10) Centrifuge at 12000rpm for 2min and pour out the waste liquid in the collection tube.Place the adsorption column at room temperature for a few minutes and allow to dry thoroughly.
Note: The purpose of this step is to remove the residual ethanol from the adsorption column, which will affect the subsequent enzymatic reaction.
(11) Put the adsorption column in a new RNase-Free centrifuge tube, add 30-50μL rnase-free Water to the middle part of the adsorption column, place at room temperature for 1min, centrifuge at 12000 RPM for 1min, collect the RNA solution, and store the RNA at -70°C to prevent degradation.

2. Reverse transcription (the tip and PCR are sterilized by damp heat without ribonuclease)

Prepare the reverse transcription reaction solution (the reaction solution is prepared on ice)

Ingredient names	Amount added
5 X Evo M-MLV RT Master Mix	2 mu L

Total RNA	-
RNase free water	Up to 10μL

Reaction conditions: 37 ° C for 15min

85 ℃ for 5 SEC

4 ℃

*1: RNA amount can be added as needed.In a 10μL reverse transcription system, a maximum of 500ng Total RNA is recommended when using the SYBR Green QPCR method;When using the probe method, a maximum of 1ug of Total RNA is recommended.

*2: Be careful to mix gently during the preparation of RT reaction solution.

3. Quantitative PCR

1) The cDNA obtained by the above reverse transcription process can be directly analyzed by quantitative PCR.Using our SYBR® Green Premix Pro Taq HS qPCR Kit (Code.AG11701) as an example, the detailed operation is as follows:

(Take ABI 7500 Real-Time PCR System as an example)

Component names	20μL system	50μL system
2X SYBR® Green Pro Taq HS Premix 10μl 25μl cDNA*1	2 mu L	5 mu L
Template*1	< 100 ng	< 200 ng
Primer F (10μM) *2	0.4 u L	1 mu L
Primer R (10μM) *2	0.4 u L	1 mu L
ROX Reference Dye (4μM) *3,4	0.4 u L	1 mu L
RNase free water	Up to 20μL	Up to 50μL

2) qPCR reaction conditions

Two-step PCR reaction procedure *1:

	The temperature	time	Number of cycles
Step 1	95 ℃	30 sec*2	1

Step 2	95 °C<br>60 °C	5 sec<br>30 sec*3	40
Step 3	Dissociation stage		

4. Process the results

ΔΔCT method:

A=CT(target gene, test sample) -CT (internal standard gene, test sample)

B=CT(target gene, control sample) -CT (internal standard gene, control sample)

K=A-B

Expression fold $=2^{-K}$

Iv. Experimental results

See Data analysis form for details of raw data and data analysis
