# Supplementary figures and images for: Identifying C1QB, ITGAM, and ITGB2 as potential diagnostic candidate genes for diabetic nephropathy using bioinformatics analysis
Source: PeerJ. 2023 May 25;11:e15437. doi: 10.7717/peerj.15437 (PMC10225123; doi:10.7717/peerj.15437)

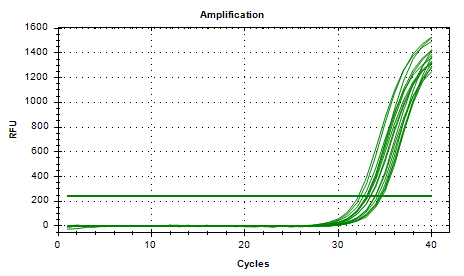

Supplement: Supplemental Information 5 — The data results of validation of hub-genes using PCR. [file peerj-11-15437-s005.zip › blood sample/1/amplification curve/C1QB.png]

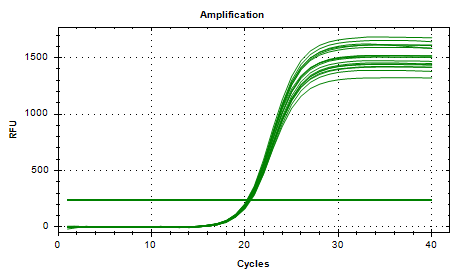

Supplement: Supplemental Information 5 — The data results of validation of hub-genes using PCR. [file peerj-11-15437-s005.zip › blood sample/1/amplification curve/GAPDH.png]

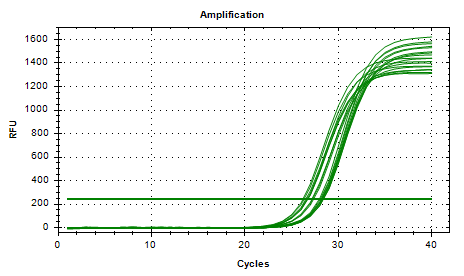

Supplement: Supplemental Information 5 — The data results of validation of hub-genes using PCR. [file peerj-11-15437-s005.zip › blood sample/1/amplification curve/ITGAM.png]

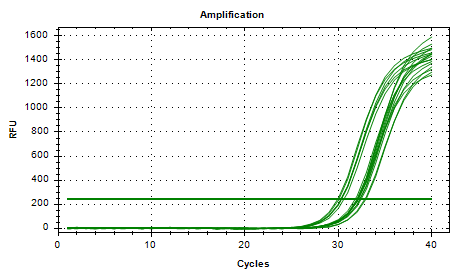

Supplement: Supplemental Information 5 — The data results of validation of hub-genes using PCR. [file peerj-11-15437-s005.zip › blood sample/1/amplification curve/ITGB2.png]

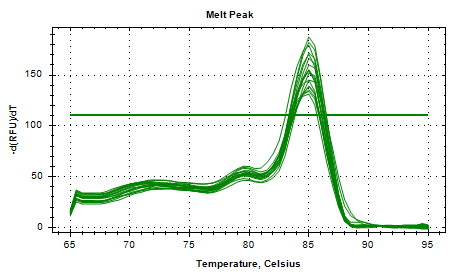

Supplement: Supplemental Information 5 — The data results of validation of hub-genes using PCR. [file peerj-11-15437-s005.zip › blood sample/1/solubility curve/C1QB.png]

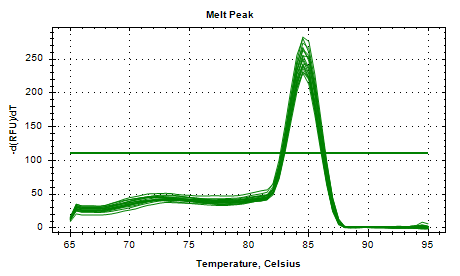

Supplement: Supplemental Information 5 — The data results of validation of hub-genes using PCR. [file peerj-11-15437-s005.zip › blood sample/1/solubility curve/GAPDH.png]

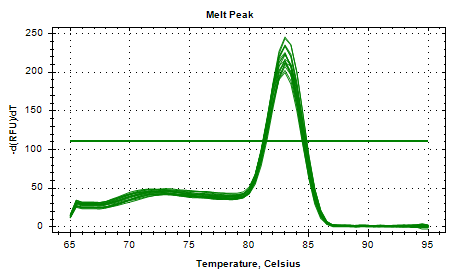

Supplement: Supplemental Information 5 — The data results of validation of hub-genes using PCR. [file peerj-11-15437-s005.zip › blood sample/1/solubility curve/ITGAM.png]

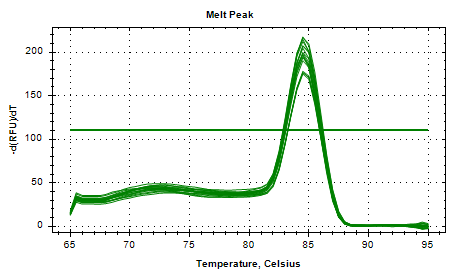

Supplement: Supplemental Information 5 — The data results of validation of hub-genes using PCR. [file peerj-11-15437-s005.zip › blood sample/1/solubility curve/ITGB2.png]

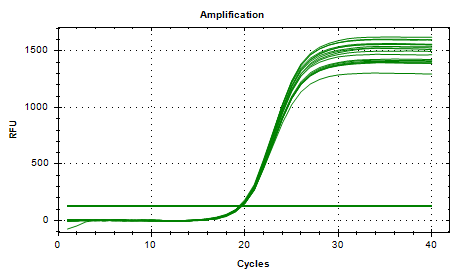

Supplement: Supplemental Information 5 — The data results of validation of hub-genes using PCR. [file peerj-11-15437-s005.zip › blood sample/2/amplification curve/GAPDH.png]

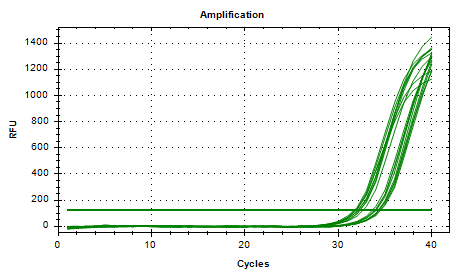

Supplement: Supplemental Information 5 — The data results of validation of hub-genes using PCR. [file peerj-11-15437-s005.zip › blood sample/2/amplification curve/IRF8.png]

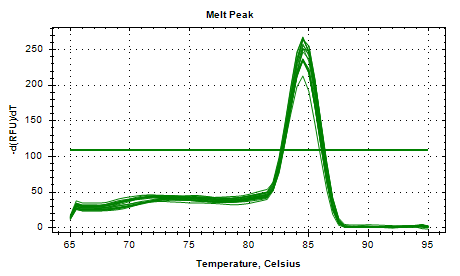

Supplement: Supplemental Information 5 — The data results of validation of hub-genes using PCR. [file peerj-11-15437-s005.zip › blood sample/2/solubility curve/GAPDH.png]

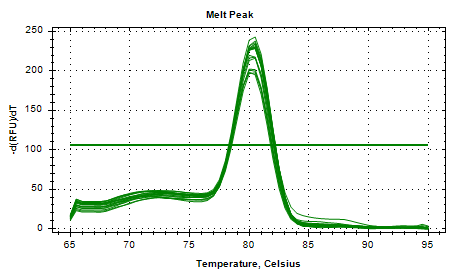

Supplement: Supplemental Information 5 — The data results of validation of hub-genes using PCR. [file peerj-11-15437-s005.zip › blood sample/2/solubility curve/IRF8.png]

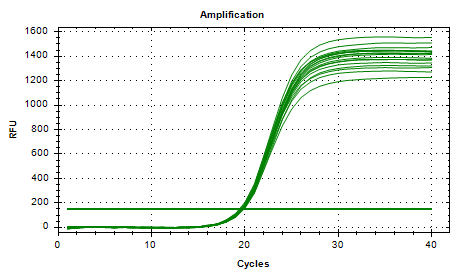

Supplement: Supplemental Information 5 — The data results of validation of hub-genes using PCR. [file peerj-11-15437-s005.zip › blood sample/3/amplification curve/GAPDH.png]

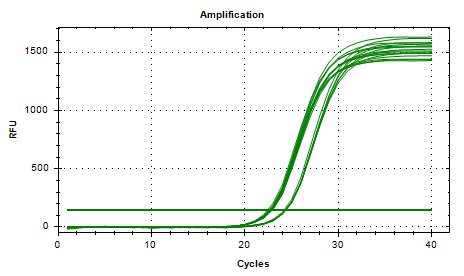

Supplement: Supplemental Information 5 — The data results of validation of hub-genes using PCR. [file peerj-11-15437-s005.zip › blood sample/3/amplification curve/HLA-DPA1.png]

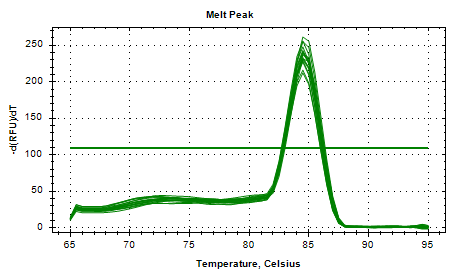

Supplement: Supplemental Information 5 — The data results of validation of hub-genes using PCR. [file peerj-11-15437-s005.zip › blood sample/3/solubility curve/GAPDH.png]

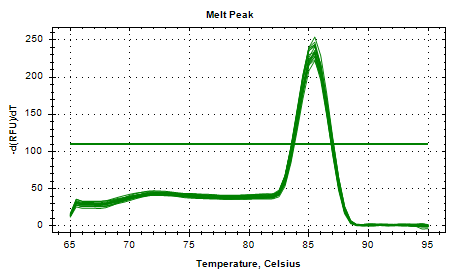

Supplement: Supplemental Information 5 — The data results of validation of hub-genes using PCR. [file peerj-11-15437-s005.zip › blood sample/3/solubility curve/HLA-DPA1.png]

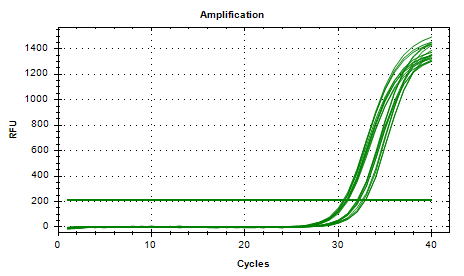

Supplement: Supplemental Information 5 — The data results of validation of hub-genes using PCR. [file peerj-11-15437-s005.zip › kidney sample/1/amplification curve/C1QB.png]

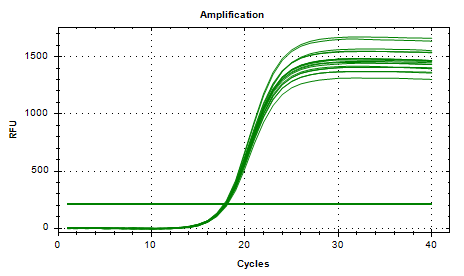

Supplement: Supplemental Information 5 — The data results of validation of hub-genes using PCR. [file peerj-11-15437-s005.zip › kidney sample/1/amplification curve/GAPDH.png]

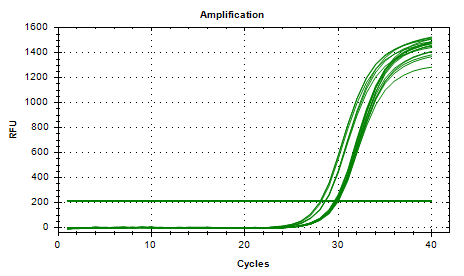

Supplement: Supplemental Information 5 — The data results of validation of hub-genes using PCR. [file peerj-11-15437-s005.zip › kidney sample/1/amplification curve/ITGB2.png]

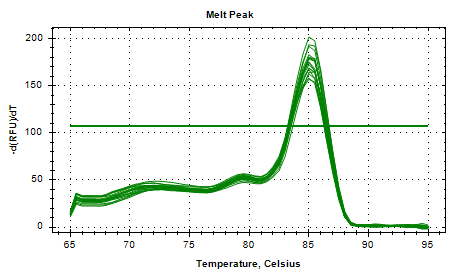

Supplement: Supplemental Information 5 — The data results of validation of hub-genes using PCR. [file peerj-11-15437-s005.zip › kidney sample/1/solubility curve/C1QB.png]

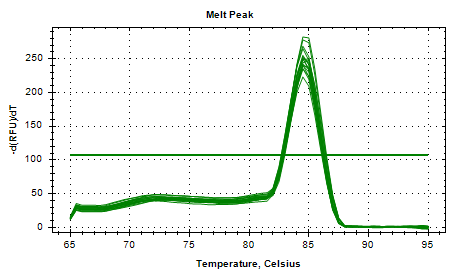

Supplement: Supplemental Information 5 — The data results of validation of hub-genes using PCR. [file peerj-11-15437-s005.zip › kidney sample/1/solubility curve/GAPDH.png]

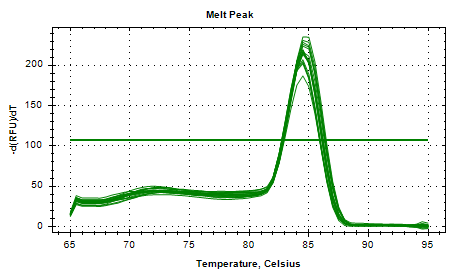

Supplement: Supplemental Information 5 — The data results of validation of hub-genes using PCR. [file peerj-11-15437-s005.zip › kidney sample/1/solubility curve/ITGB2.png]

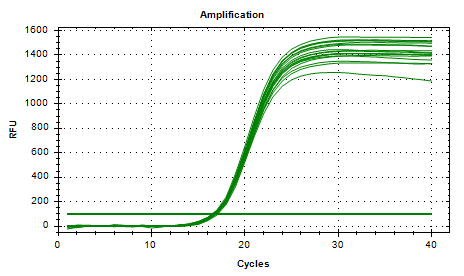

Supplement: Supplemental Information 5 — The data results of validation of hub-genes using PCR. [file peerj-11-15437-s005.zip › kidney sample/2/amplification curve/GAPDH.png]

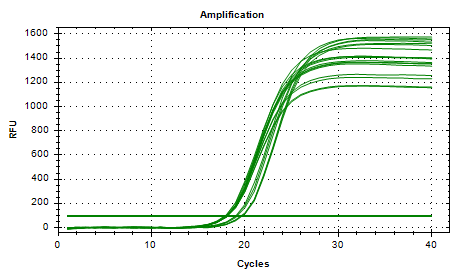

Supplement: Supplemental Information 5 — The data results of validation of hub-genes using PCR. [file peerj-11-15437-s005.zip › kidney sample/2/amplification curve/HLA-DPA1.png]

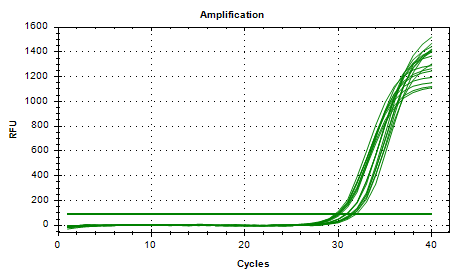

Supplement: Supplemental Information 5 — The data results of validation of hub-genes using PCR. [file peerj-11-15437-s005.zip › kidney sample/2/amplification curve/IRF8.png]

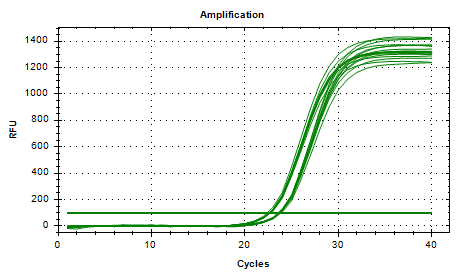

Supplement: Supplemental Information 5 — The data results of validation of hub-genes using PCR. [file peerj-11-15437-s005.zip › kidney sample/2/amplification curve/ITGAM.png]

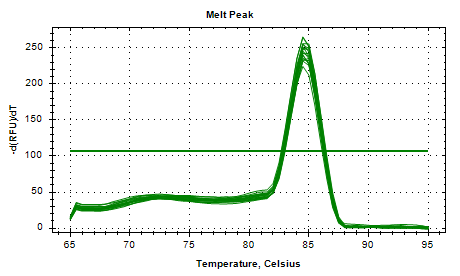

Supplement: Supplemental Information 5 — The data results of validation of hub-genes using PCR. [file peerj-11-15437-s005.zip › kidney sample/2/solubility curve/GAPDH.png]

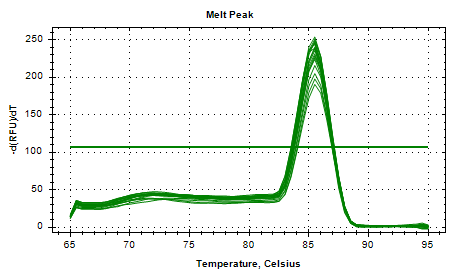

Supplement: Supplemental Information 5 — The data results of validation of hub-genes using PCR. [file peerj-11-15437-s005.zip › kidney sample/2/solubility curve/HLA-DPA1.png]

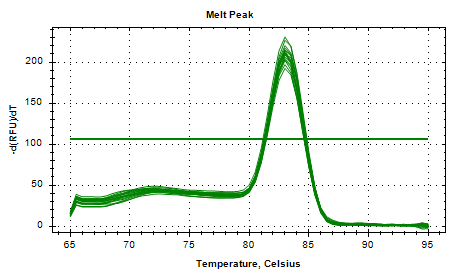

Supplement: Supplemental Information 5 — The data results of validation of hub-genes using PCR. [file peerj-11-15437-s005.zip › kidney sample/2/solubility curve/ITGAM.png]

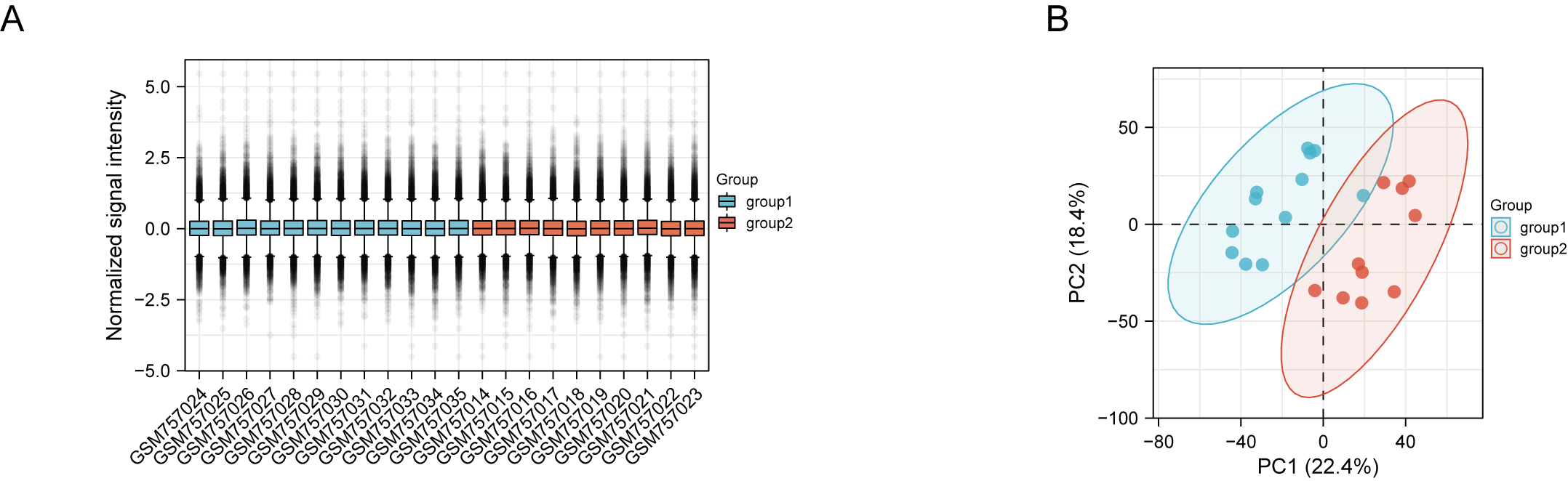

Supplement: Supplemental Information 7 — Red and blue spots represent samples from DN and control group, namely. [file peerj-11-15437-s007.png]

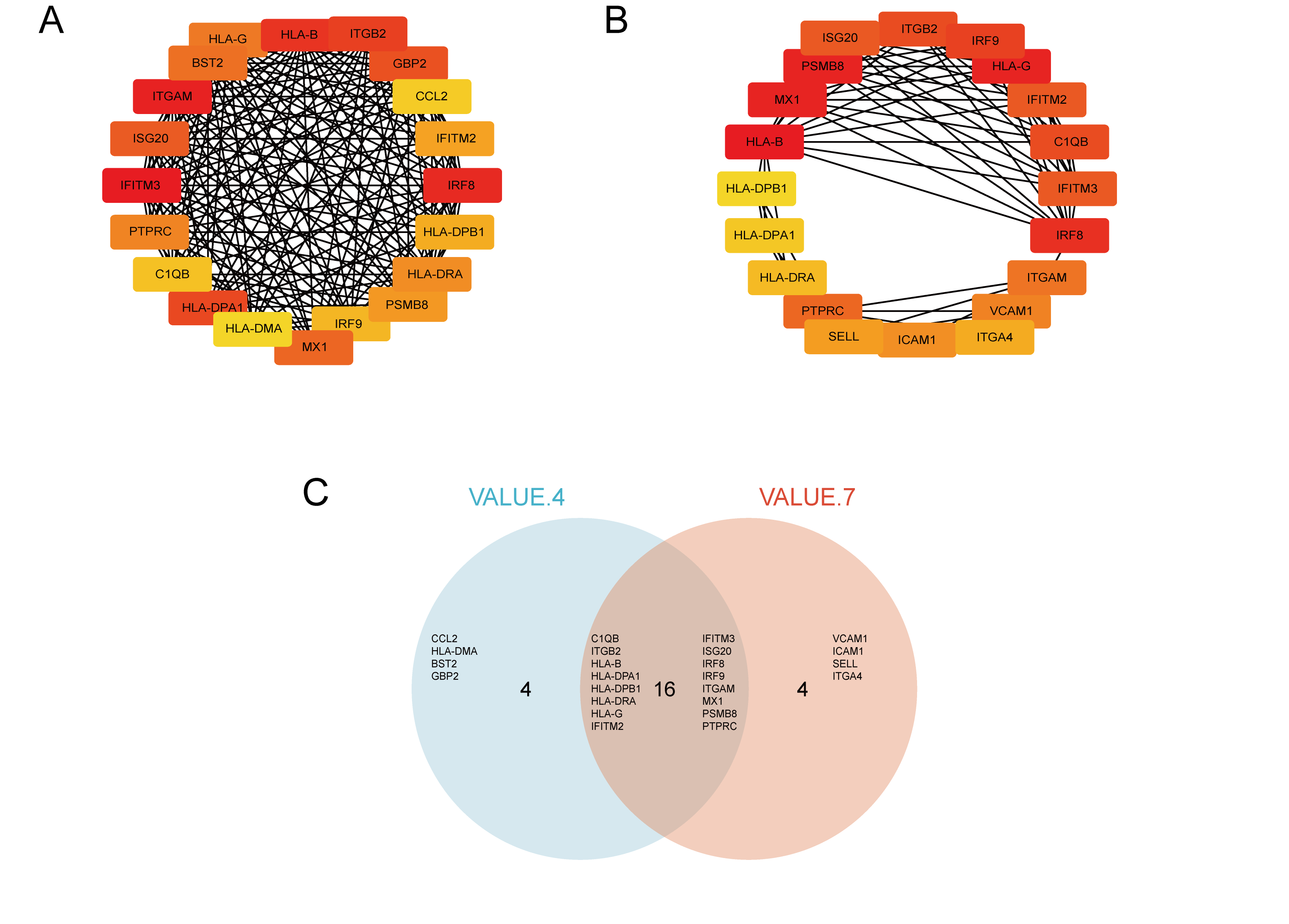

Supplement: Supplemental Information 8 [file peerj-11-15437-s008.png]

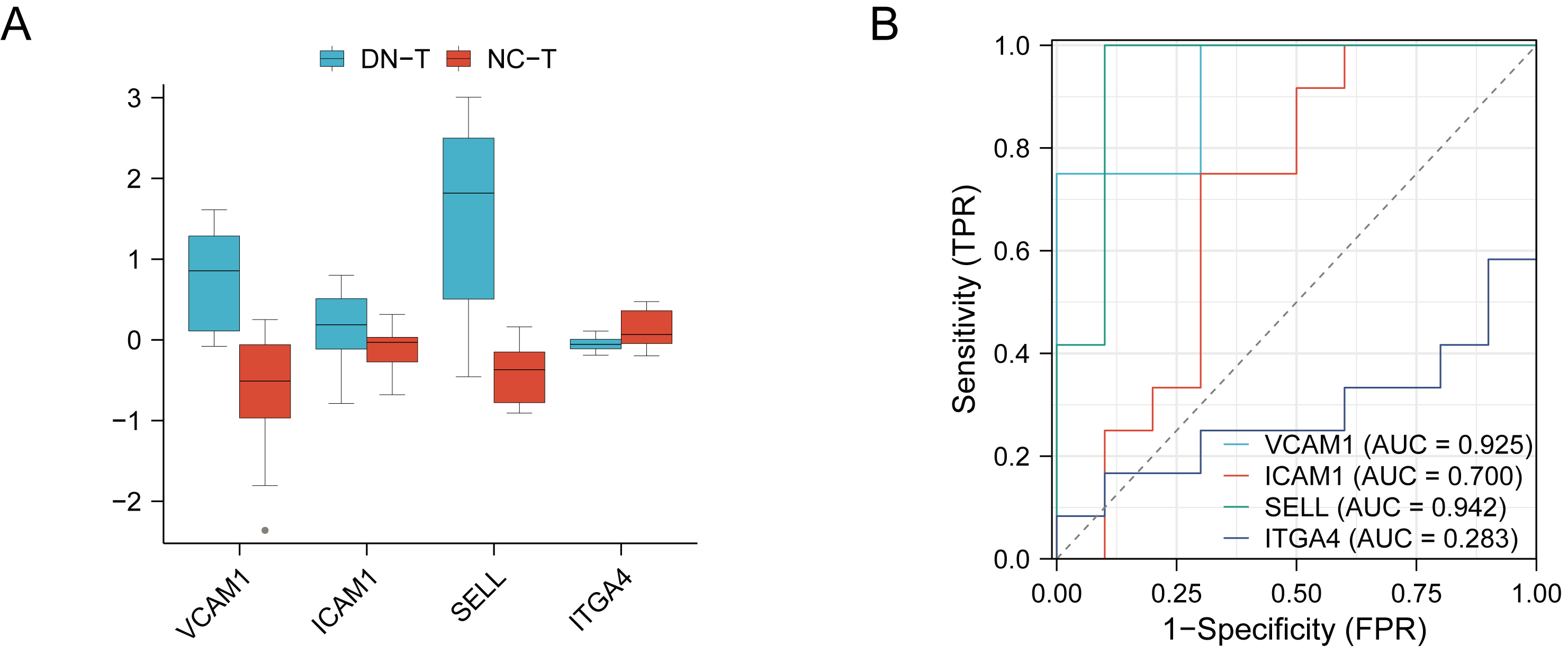

Supplement: Supplemental Information 9 [file peerj-11-15437-s009.png]

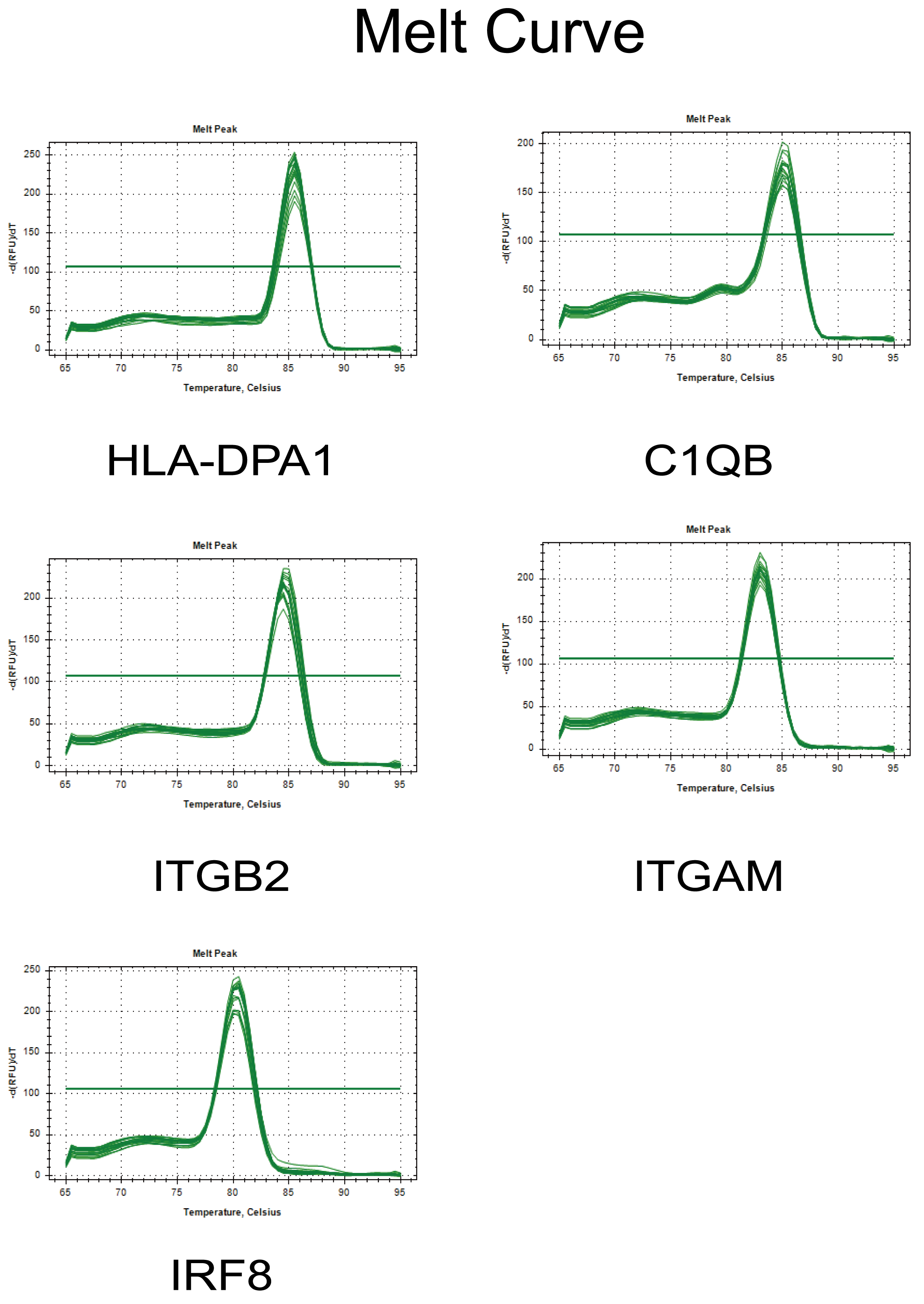

Supplement: Supplemental Information 10 [file peerj-11-15437-s010.png]
